# Long-term effectiveness of the Mindful Self-Compassion programme compared to a Mindfulness-Based Stress Reduction intervention: a quasi-randomised controlled trial involving regular mindfulness practice for 1 year

**DOI:** 10.3389/fpsyg.2025.1597264

**Published:** 2025-04-28

**Authors:** Antonio Crego, José Ramón Yela, María Ángeles Gómez-Martínez, Elena Sánchez-Zaballos, Aitor Vicente-Arruebarrena

**Affiliations:** ^1^Department of Psychology, Pontifical University of Salamanca, Salamanca, Spain; ^2^Clinical and Health Psychology Service, Pontifical University of Salamanca, Salamanca, Spain

**Keywords:** Mindful Self-Compassion, Mindfulness-Based Stress Reduction, longitudinal research, quasi-RCT, mental health, psychological flexibility, anxiety, depression

## Abstract

**Introduction:**

This study compares the Mindful Self-Compassion (MSC) programme with the Mindfulness-Based Stress Reduction (MBSR), a well-established intervention, and a control group, and includes 1 year of continuous practice.

**Methods:**

A longitudinal quasi-RCT was conducted with measurements at baseline, post-training, 6 months and 1 year. A total of 170 individuals (75.9% female) were randomly assigned to the MSC (*n* = 48) and MBSR (*n* = 65) groups, with a non-fully randomised wait-list CG (*n* = 57).

**Results:**

Using intention-to-treat (ITT) and per-protocol (PP) analysis strategies, results consistently indicated that standard 8-week MSC and MBSR trainings produced benefits on anxiety, depression, perceived stress, and positive and negative affect, as well as on variables related to psychological flexibility, compared with CG. These gains were maintained over a year of continuous practice in both training groups. Overall, the two programmes, MSC and MBSR, showed a similar trajectory over the measurement periods.

**Discussion:**

The 8-week MSC programme and the regular practice of mindfulness and self-compassion appear to be an effective intervention for promoting mental health in the general population, with benefits similar to those derived from the practice of exercises from well-known mindfulness programmes such as MBSR.

## Introduction

People who face challenging situations that can lead to psychological distress often also face structural, cultural, economic, and health barriers that contribute to the mental health treatment gap (Carbonell et al., 2020). These intersecting issues highlight the critical need for comprehensive strategies that not only expand access to mental health services but also prioritise long-term support and prevention (Kirkbride et al., 2024; Singh et al., 2022). Within this framework, nurturing a proactive approach to mental health involves equipping individuals with emotional self-care skills. Mindfulness programmes may represent a promising avenue to achieve this objective (Galante et al., 2021). However, while numerous studies have demonstrated the short-term benefits of Mindfulness-based interventions (MBIs) like the Mindfulness-Based Stress Reduction programme (MBSR) (Kabat-Zinn, 1990) and Mindfulness-Based Cognitive Therapy (MBCT) (Segal et al., 2002) programmes, fewer have investigated the Mindful Self-Compassion (MSC) protocol (Germer and Neff, 2019) specifically, and almost none have assessed the durability of effects beyond 3 months. In this regard, this study evaluates whether the MSC programme, compared to the established MBSR protocol and a waitlist control, produces lasting improvements in mental health indicators and psychological flexibility over 1 year of guided practice.

MBIs aim at cultivating a mindful disposition in daily life. Mindfulness is a process and a state of “awareness that emerges when we intentionally pay attention to the present moment, without judging or evaluating that experience moment to moment” (Kabat-Zinn, 1990, 2003). In the last decade, scientific evidence has supported the relationship between the practice of mindfulness-based interventions (MBIs) and psychological variables. These findings have promoted a significant increase in research focused on preventing and improving health issues (see McClintock et al., 2019). In this regard, a meta-analysis reviewing 49 studies in non-clinical samples, found that MBSR and MBCT programmes were associated with benefits in psychological health and wellbeing in non-clinical populations (Querstret et al., 2020). The authors suggest that both MBSR and MBCT could be used preventively to reduce symptoms associated with poor mental health (depression, anxiety, burnout, fatigue, stress) and increase positive indices of mental health (improved quality of life or life satisfaction). As pointed out by Judd et al. (2002), much of the burden of problems in the population is due to subclinical symptoms of mental health disorders, rather than diagnosed mental health disorders. People with subclinical mental health symptoms are more likely to develop diagnosable mental health problems in the future. Access to such programmes could play an important role in preventing the onset of mental health problems (Sadek and Bona, 2000).

Another type of meditation, called loving-kindness and compassion (LKCM), is receiving increasing attention (Zeng et al., 2023). The integration of LKCM with MBIs is one of the essential features of so-called second-generation mindfulness-based interventions (Van Gordon et al., 2015). Such programmes aim to develop not only an attitude of mindfulness towards our own emotional experiences (i.e., an attitude of open acceptance of them), but also to develop a benevolent attitude of continuous non-self-criticism towards ourselves, as well as an attitude of openness to suffering, as a consubstantial part of life that is common to all human beings. One such training is the Mindfulness Self-Compassion (MSC) programme developed by Germer and Neff (2013, 2019) and Neff and Germer (2013, 2018).

### LKCM and psychological benefits in short-term studies

Previous research has found strong evidence for the effectiveness of mindfulness-based interventions, such as MBSR and MBCT (Jiménez-Gómez et al., 2022; McClintock et al., 2019; Querstret et al., 2020). Moreover, MBSR and MBCT trainings have shown efficacy in the treatment of various mental disorders (Australian Psychological Society, 2018). Concerning the Mindful Self-Compassion training (MSC), a growing body of literature reports that this training is associated with improvements in wellbeing (Neff and Germer, 2017; Yela et al., 2020a; Yela et al., 2022) and reductions in psychopathological symptoms (Friis et al., 2016; Jiménez-Gómez et al., 2022). However, research specifically focused on the MSC programme is still scarce.

LKCM programmes produce changes in a wide range of variables related to psychological wellbeing and mental health. For instance, a review by Graser and Stangier (2018) concluded that compassion-based interventions were effective in treating psychotic disorders, affective disorders, major depressive disorder, eating disorders and patients with suicidal ideation in the past year. Loving-kindness meditation was effective in treating chronic pain, and a combination of both was effective in borderline personality disorder. Focusing specifically on the anxiety variable, the recent meta-analysis by Zheng et al. (2023) showed that LKCM interventions were effective in producing significant decreases, with small to medium effect sizes. A meta-analysis by Wilson et al. (2019) included programmes in which self-compassion components were present and found improvements in anxiety and depression with small effect sizes. The meta-analysis by Lv et al. (2024) reviews the efficacy of programmes including loving kindness, compassion, appreciative joy and equanimity and finds support for the effectiveness of these programmes on depressive symptoms, with intermediate/high effect sizes depending on whether they were controlled or uncontrolled trials.

Interestingly, some meta-analyses have studied the impact of LKCM interventions beyond concrete symptoms to include other psychological variables. Kirby et al. (2017) reviewed 21 RCT trials using compassion-based interventions. They found significant changes in compassion, self-compassion, mindfulness, depression, anxiety, psychological distress and wellbeing, with medium effect sizes.

In a meta-analysis that included 27 RCT studies in general and clinical populations, Ferrari et al. (2019) found that self-compassion training improved levels of mindfulness and self-compassion skills and reduced stress, self-criticism, anxiety and depressive symptoms, with medium effect sizes. Only seven of these studies included follow-up (ranging from 1 to 3 months). Small but significant effects were shown at follow-up for depressive symptoms, while maintenance of improvement in self-compassion was not significant. Han and Kim (2023) increased the sample of papers reviewed by Ferrari et al. (2019) to include 56 studies, supporting the previous results: self-compassion-focused programmes produced significant improvements (pre-post changes) in stress and depression with a medium effect size, and improvements in anxiety levels with a small effect size.

Finally, Gu et al. (2022) conclude that the effects of LKCM programmes (duration between 3.5 and 10 weeks) also produce significant improvements in life satisfaction in pre-post designs.

### Variables involved in the effects of LKCM programmes

A variety of mechanisms have been suggested to account for the effects of mindfulness on mental health, such as decentering, value clarification, exposure, psychological flexibility, self-management skills (Brown et al., 2015), attention monitoring, acceptance of internal experiences (Lindsay and Creswell, 2017), and the development of self-compassionate attitudes (Gu et al., 2015).

Some of these variables are part of the construct of ‘psychological flexibility’ formulated by Hayes et al. (1999), which is key in Acceptance and Commitment Therapy (ACT). In addition, authors like Neff and Tirch (2013) have pointed out the interest of investigating the relationship between self-compassion and constructs such as acceptance, perspective-taking and psychological flexibility (mindfulness, defusion, observing-self, acceptance, value clarification and engaged action/behavioural activation). In addition, Germer (2025) has argued that self-compassion is a transtheoretical and transdiagnostic process, present in numerous psychotherapeutic interventions. Recently, Hayes (2025) himself has proposed that self-compassion can be considered as a transdiagnostic process closely related in clinical interventions to psychological flexibility in a continuum of mindfulness, acceptance and self-compassion.

Empirical research has also connected self-compassion and psychological flexibility-related variables. For instance, self-compassion, perceived meaning in life, and experiential avoidance may explain the relationship between mindfulness meditation and mental health (Yela et al., 2020b), and decreased experiential avoidance may explain the changes in anxiety, depression and wellbeing following MSC training in a community sample (Yela et al., 2022).

Recently, attempts have also been made to link self-compassion and psychological flexibility from a theoretical perspective. In this sense, Yela and Crego (2025) have proposed that the increase in psychological flexibility and the reduction of experiential avoidance may be mechanisms that could explain the benefits derived from self-compassion practices. Similarly, Weinstein (2025) has proposed a theoretical integration between self-compassion practices and Acceptance and Commitment Therapy, a third wave cognitive-behavioural therapy. His approach maps self-compassion onto the psychological model of flexibility, suggesting that ‘tender self-compassion’ practices support the development of ‘acceptance’ and ‘defusion’ processes, whereas the use of ‘fierce self-compassion’ practices supports the development of ‘value clarification’ and ‘committed action’ processes.

### Longitudinal studies

To our knowledge, no studies involving long-term regular practitioners of LKCM or MBI standard protocols have been published yet. Only, the European Medit-Ageing Project focus on the long-term effects (18 months) of mindfulness, loving kindness and self-compassion meditation practices on the mental health and wellbeing of older people (Poisnel et al., 2018; Chételat et al., 2022). The differences between the treatment and active control groups after 18 months indicated statistically significant changes in two variables that included components of mindfulness and self-compassion, i.e., attentional regulation skills and social–emotional skills (but not in self-awareness skills).

### The present study

The efficacy of the MBSR programme has been widely researched, and it can be considered a “well-established protocol.” As we have pointed out, in the field of LKCM interventions, efficacy studies have been conducted on programmes that combine a variety of techniques from various mindfulness, (self-) compassion and self-kindness trainings. However, research on the efficacy of a standardised programme, specifically focused on self-compassion skills, such as the MSC, remain scarce. In addition, research on regular mindfulness practice and self-compassion over time to identify possible psychological effects has been even less analysed and represents a gap in the field.

In this context, the present study aims to analyse the effects of MSC training and regular MSC practice on mental health and wellbeing-related variables connected with psychological flexibility (e.g., mindfulness, self-compassion, presence of meaning in life, cognitive defusion, acceptance of inner experiences, behaviour activation) compared to an active treatment MBSR group and to a waitlist CG. Specifically, this research aims to, first, assess the 8-week MSC training efficacy to produce beneficial outcomes on mental health, wellbeing and psychological flexibility-related variables, compared to a well-established 8-week training (MBSR) and a CG. Second, this research will identify the effects over time (up to 12-month follow-up) of the regular practice of MSC exercises on these psychological variables, compared to regular practice of MBSR exercises and to a waitlist CG. To our knowledge, this is the first quasi-randomised study to assess the comparative effectiveness of MSC and MBSR with sustained practice over 1 year.

## Methods

### Sample

The participants were 170 individuals (75.9% female), with a mean age = 42.81 years (*Sd* = 15, 51; *min.* = 18, *max.* = 65). Regarding their educational level, 1.8% of the participants had basic education; 13.5% had professional training or attended high school; 55.3% had undergraduate university studies; and 29.4% reached postgraduate level. In relation to their employment status, most of the participants were working (61.2%); 15.9% were students; 14.7% were unemployed; 7.6% retired; 0.6% in other situations (e.g., functional disability).

The MSC group consisted of 48 participants (79.2% female), with a mean age = 45.31 (*Sd* = 14.45; *min* = 18, *max* = 64). Most of the MSC group members had undergraduate (23 participants; 47.9%) or postgraduate (16 participants; 33.3%) university studies; 8 (16.7%) had completed professional training or high school; and 1 (2.1%) had basic studies. Regarding their employment status, 32 (66.7%) participants in the MSC group were employed; 3 (6.3%) were students; 4 (8.4%) were retired; 8 (16.7%) were unemployed and 1 (2.1%) reported functional disability.

The MBSR group comprised 65 participants (69.2% female), with a mean age = 48.54 (*Sd* = 13.05; *min.* = 18, *max.* = 64). The MBSR group members had mostly undergraduate (35 participants; 53.8%) or postgraduate (21 participants; 32.3%) university studies; 9 (13.8%) had completed professional training or high school. Regarding their employment status, 48 (73.8%) participants in the MBSR group were in working; 5 (7.7%) were students; 7 (10.7%) were retired; and 5 (7.7%) were unemployed.

The CG included 57 participants (80.7% female), with a mean age = 34.18 (*Sd* = 15.38; *min.* = 18, *max.* = 65). The CG members had mainly undergraduate (36 participants; 63.2%) or postgraduate (13 participants; 22.8%) university studies; 6 (10.5%) had completed professional training or high school; and 2 (3.5%) had basic education. Regarding their employment status, 24 (42.1%) GC participants were in active service; 19 (33.3%) were students; 2 (3.5%) were retired; and 12 (21.1%) were unemployed.

### Design and procedure

Participants were recruited through advertisements in various local media in the city of Salamanca (Spain), as well as through posts on social networks during November and December of 2022. The candidates received detailed information about the study and the commitments that participation entailed (i.e., attendance to training sessions in the case of experimental conditions, answering questionnaires, biological analysis—not reported in this study—) in two face-to face group meetings held in January 2023. In addition, they were informed about the authorisation of the Research Ethics Committee of the Pontifical University of Salamanca (CEI 07/22/2019), the treatment of personal data, and protection and use of their data. Informed consent was obtained from all the participants. The evidence-based CONSORT Statement recommendations for conducting and reporting randomised trials was followed. This study was registered at ClinicalTrials.gov (ID: NCT05695586).

Inclusion criteria included being healthy individuals between 18 and 65 years old, with no previous practice or knowledge of mindfulness techniques. This study was part of a larger research project involving biological measures collected by means of blood analyses, and therefore we used exclusion criteria comparable to those used in studies with similar samples and biomarkers collection (e.g., Alda et al., 2016; Pace et al., 2013). Exclusion criteria were having suffered or currently suffering from a psychiatric disorder, being currently in psychiatric or psychological treatment, suffering from a severe medical disorder that could affect inflammatory response, as well as systemic inflammation (cancer, AIDS or any other chronic disease that occurs with inflammation, including COVID-19), having received psychotropic medication within 2 weeks prior to blood extractions, or having signs of acute infection on the day of the blood extraction.

**Figure 1:**
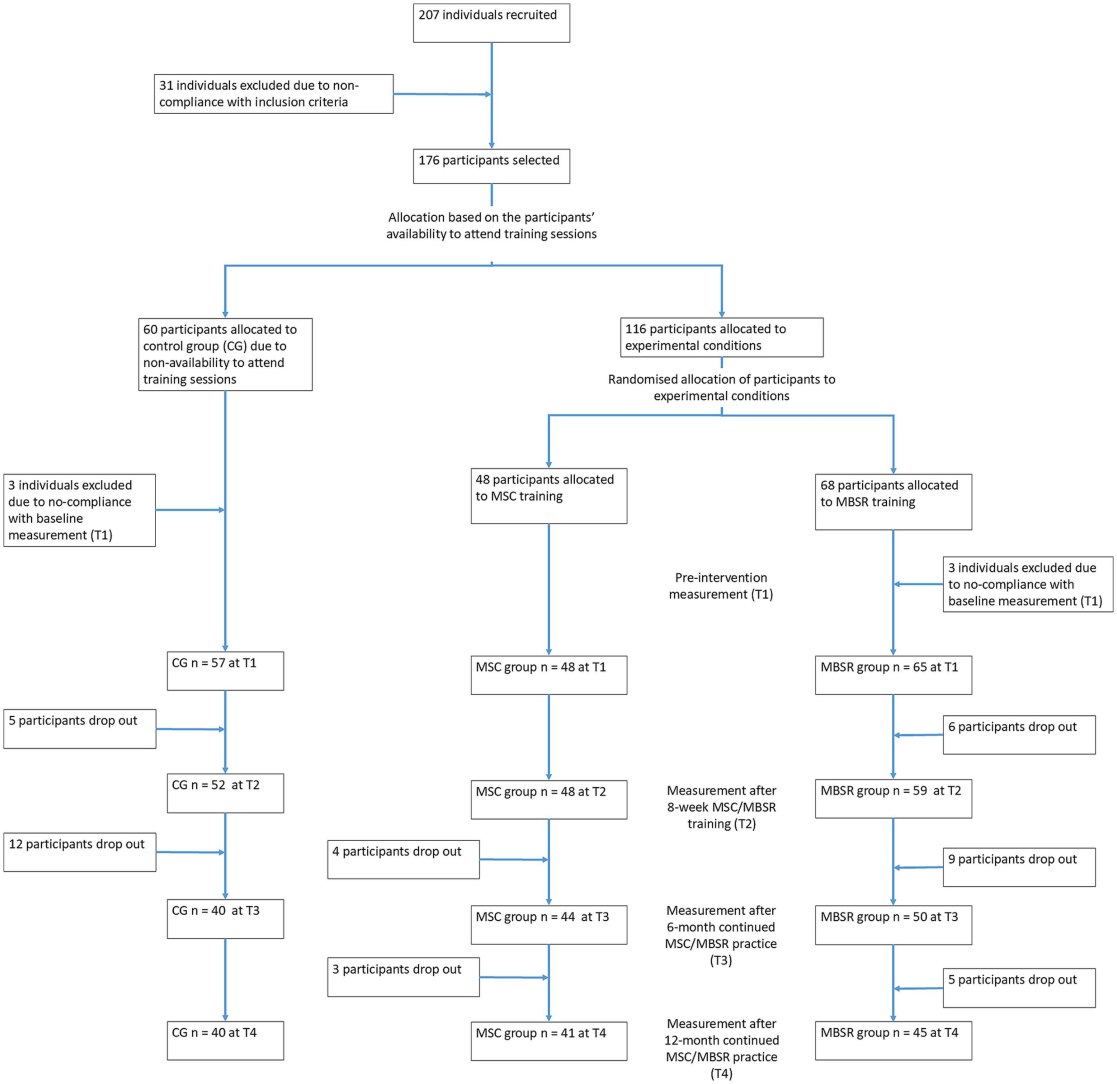
Flowchart of participants allocation.

A longitudinal quasi-randomised controlled trial (quasi RCT) with three groups (MSC training, MBSR training, control group) and measurements at pre- (T1), post-intervention (T2), 6-month follow-up (T3), and 1-year follow-up was conducted (T4). Eligible candidates who met inclusion criteria and confirmed their availability for the training were randomly allocated to MSC or MBSR groups. The 8-week MSC and MBSR trainings started in February 2023. Participants were blinded to whether they were receiving an MSC or MBSR intervention, and only knew that they were receiving mindfulness-based training to promote wellbeing and emotional self-care. A computer-based simple randomisation procedure was used. Participants who reported limitations to attending training groups (e.g., due to work commitments, scheduling problems, etc.) were assigned to CG. Figure 1 shows the flowchart of participant allocation and attrition over time.

Participants in the MSC group were trained in the standard 8-week Mindfulness Self-Compassion (MSC) protocol (Germer and Neff, 2019; Neff and Germer, 2018). Participants in the MBSR group received the 8-week Mindfulness-Based Stress Reduction training as described by Stahl and Goldstein (2019). An outline of the protocol used can be found in Stahl and Goldstein (2019), Chapter 11 (pp. 203–206), which provides detailed guidance on the content of each session and how to practice each week. Both MSC and MBSR trainings include formal (i.e., meditation sessions) and informal (i.e., exercises that are carried out throughout the day) practices. Each weekly session of both MSC and MBSR lasted 2.5 h during the 8-week standard training phase. In addition, both trainings included an intensive practice day (i.e., ‘retreat’) of 5 h. The MBSR and MSC trainings also required participants to perform between-session tasks, usually consisting of practicing formal meditation skills already learned and doing informal practices. Once participants completed the MSC or MBSR 8-week trainings they entered a 12-month phase of regular supervised practice, in which they continued to perform MSC or MBSR exercises, as applicable, on a guided basis. Supervised practice was provided on a weekly basis, with 1 h-sessions.

The MSC and MBSR trainings were provided at the facilities of the [BLINDED NAME OF INSTITUTION] for Pontifical University of Salamanca. Two MSC groups and three MBSR groups were simultaneously delivered. A clinical psychologist accredited as *MSC Certified Teachers* by the University of California and the Center for Mindful Self-Compassion (San Diego, CA, USA) trained the MSC groups and provided the subsequent supervised practice. Three health psychologists led the corresponding 8-week MBSR sessions and the 12-month supervised continued practice sessions. All MBSR trainers had in-depth knowledge of the programme and had previously participated in it, with more than a year’s experience of continuous personal mindfulness practice. In addition, the trainers had taught the MBSR programme at postgraduate level, conducted or supervised doctoral research on the application of the MBSR programme, and had extensive experience in mindfulness research. The instructors had the official accreditation required in Spain for the practice of health psychology.

The waitlist CG had no previous knowledge and/or practice with MBSR/MSC and did not receive the either training. People in the CG were waitlisted to receive a MBSR or MSC training once the 12-month intervention was finished.

The participants were contacted via email in advance at each measurement moment, i.e., pre- (T1), post-intervention (T2), 6-month (T3), and 12- month (T4) continued practice and data were gathered through an online questionnaire survey. All participants received 30€ at each measurement for motivation purposes.

### Instruments

*Psychological flexibility*. Psychological flexibility was measured using the Psy-Flex scale (Gloster et al., 2021; Spanish translation by Ruiz Jiménez et al., n.d.). This scale uses 6 items scored from 1 (never) to 5 (very often) on a Likert-type scale. Total scores are obtained for each participant, with higher scores representing a higher level of psychological flexibility. Cronbach’s alpha internal consistency ranged from 0.842 (T1) to 0.894 (T4) in the intervention groups, and from 0.816 (T1) to 0.906 (T4) in the control group.

*Self-compassion*. The Spanish version of the 26-item Self-Compassion Scale (SCS; Garcia-Campayo et al., 2014) was used. This scale measures the construct of self-compassion as defined by Neff (2003) where self-compassion entails being kind towards oneself, seeing one’s experiences as part of the larger human condition, and being mindfully aware of one’s inner experiences, instead of being unkindly, self-critical, feeling isolated or strange, and over-identification with painful thoughts and feelings. Participants respond on a 5-points Likert-type scale, from 1 (Never) to 5 (Always). Total scores are calculated by averaging each participant’s responses to the items (range, 1–5), with higher scores representing greater self-compassion. Cronbach’s alpha internal consistency ranged from 0.919 (T2) to 0.950 (T4) in the intervention groups, and from 0.894 (T1) to 0.939 (T3) in the control group.

*Mindfulness*. The Mindful Attention Awareness Scale (MAAS; Brown and Ryan, 2003; Soler Ribaudi et al., 2012) was used. This scale comprises 15 items designed to measure the individual’s general mindfulness ability. It uses a 6-point Likert-type response format from 1 (Almost always) to 6 (Almost never). Total scores are obtained by averaging each participant’s responses to the items, with higher scores meaning a greater mindfulness capacity. Cronbach’s alpha internal consistency ranged from 0.842 (T1) to 0.894 (T4) in the intervention groups, and from 0.816 (T1) to 0.906 (T4) in the control group.

*Meaning in life*. The Presence of Meaning 5-item subscale from the Meaning in Life Questionnaire (MLQ) (Steger et al., 2006) was used to assess how much respondents feel their lives have meaning. Respondents answer each item on a 7-point Likert-type scale ranging from 1 (Absolutely True) to 7 (Absolutely Untrue). Higher scores represent experiencing higher levels of meaning in life. Cronbach’s alpha internal consistency ranged from 0.859 (T1) to 0.909 (T3) in the intervention groups, and from 0.846 (T3) to 0.911 (T2) in the control group.

*Cognitive fusion*. The Cognitive Fusion Questionnaire (CFQ) (Gillanders et al., 2014; Romero-Moreno et al., 2014) uses 7 items scored from 1 (never) to 7 (always) on a Likert-type scale. Total scores are obtained for each participant by averaging his/her responses to the items, with higher scores representing a higher level of cognitive fusion. Cronbach’s alpha internal consistency ranged from 0.901 (T3) to 0.931 (T2) in the intervention groups, and from 0.905 (T4) to 0.915 (T2) in the control group.

*Experiential avoidance*. The Acceptance and Action Questionnaire-II (AAQ-II) (Bond et al., 2011; Ruiz et al., 2013) was used. This scale comprises 7 items intended to measure experiential avoidance, that is, an individual’s unwillingness to be exposed to and accept difficult inner experiences (e.g., thoughts, feelings, sensations) even when doing so leads to behaving in a manner that could be inconsistent with one’s values and goals. Participants rate each item on a 7-point Likert-type scale, from 1 (Never true) to 7 (Always true). Total scores are obtained for each participant by averaging the responses to the items, with higher scores representing a higher level of experiential avoidance. Cronbach’s alpha internal consistency ranged from 0.850 (T2) to 0.891 (T4) in the intervention groups, and from 0.849 (T2) to 0.918 (T4) in the control group.

*Behaviour activation*. The Activation Subscale of the Behavioural Activation for Depression Scale (BADS) scores (Kanter et al., 2007; Barraca et al., 2011) was used. The 7-item “Activation” subscale of the Behavioural Activation for Depression Scale (BADS) measures focused, goal-directed activation and completion of planned activities, e.g., “I did things even though they were hard because they fit in with my long-term goals for myself.” Responses are made on a 7-point Likert-type scale from 0 (Not at all) to 6 (Completely). Items were averaged to obtain a total score for each participant. Higher scores reflect a higher level of behavioural activation. Cronbach’s alpha internal consistency ranged from 0.854 (T1) to 0.924 (T4) in the intervention groups, and from 0.868 (T4) to 0.927 (T2) in the control group.

*Depression and anxiety*. Depression and anxiety were measured using the Hospital Anxiety and Depression Scale (HAD-S), (Zigmond and Snaith, 1983; Terol Cantero et al., 2007). This scale comprises 14 items intended as a screening instrument to detect possible anxiety (7 items) and depression (7 items). Examples items are “I get sudden feelings of panic” (anxiety) and “I have lost interest in my appearance” (depression). Participants respond by selecting 1 of 4 alternatives that are scored from 0 to 3. Scores for anxiety and depression are calculated by adding the individual’s responses to the items in each subscale, with higher scores indicating higher levels of anxiety and depression. For the anxiety subscale, Cronbach’s alpha internal consistency ranged from 0.737 (T3) to 0.827 (T1) in the intervention groups, and from 0.827 (T1) to 0.887 (T3) in the control group. For the depression subscale, Cronbach’s alpha internal consistency ranged from 0.719 (T3) to 0.826 (T1) in the intervention groups, and from 0.701 (T2) to 0.857 (T4) in the control group.

*Stress*. The Perceived Stress Scale (PSS) (Cohen et al., 1983; Remor and Carrobles, 2001) was used. This scale uses 14 items scored from 0 (never) to 4 (very often) on a Likert-type scale. Total scores are obtained for each participant, with higher scores representing a higher level of perceived stress. Cronbach’s alpha internal consistency ranged from 0.882 (T3) to 0.904 (T4) in the intervention groups, and from 0.824 (T2) to 0.891 (T4) in the control group.

*Positive and negative affect*. The Positive and Negative Affect Schedule (PANAS) (Watson et al., 1988; López-Gómez et al., 2015) scale was used. This instrument consists of 10 items representing positive moods (e.g., interested, enthusiastic, inspired) and 10 items representing negative moods (e.g., irritable, upset, afraid). Participants are asked to rate the extent to which they had recently experienced each of the 20 feelings or emotions on a 5-point Likert-type scale, ranging from 1 (Very slightly or not at all) to 5 (Extremely). Two separate total scores corresponding to positive and negative affect are obtained for each participant. Total scores (ranging from 1 to 5) are calculated by averaging each respondent’s answers to the 10 items included in the positive/negative affect scales, with higher scores indicating experiencing more positive/negative moods. For the Positive Affect subscale, Cronbach’s alpha internal consistency ranged from 0.890 (T4) to 0.908 (T1) in the intervention groups, and from 0.860 (T3) to 0.902 (T1) in the control group. For the Negative Affect subscale, Cronbach’s alpha internal consistency ranged from 0.899 (T1) to 0.936 (T4) in the intervention groups, and from 0.857 (T2) to 0.924 (T3) in the control group.

*Happiness*. The Subjective Happiness Scale (Lyubomirsky and Lepper, 1999; Extremera and Fernández-Berrocal, 2014) was used. The Subjective Happiness Scale (SHS) is a 4-item scale used to measure the global level of perceived happiness. All items use a 7-point Likert-type scale. The total scores of subjective happiness are calculated for each participant by averaging responses to the 4 items (range 1–7), with higher scores indicating greater subjective happiness. Cronbach’s alpha internal consistency ranged from 0.796 (T3) to 0.876 (T4) in the intervention groups, and from 0.845 (T1) to 0.879 (T3) in the control group.

*Satisfaction with life*. The Satisfaction with Life Scale (Diener et al., 1985; Atienza et al., 2003). The SWLS consists of 5 items representing statements indicative of contentment with one’s life and its conditions. The response format is a 7-point Likert-type scale, ranging from 1 (Strongly disagree) to 7 (Strongly agree). The scale’s total scores are calculated by averaging answers to the 5 items, with higher scores (ranging from 1 to 7) indicating greater satisfaction with life. Cronbach’s alpha internal consistency ranged from 0.817 (T2) to 0.884 (T1) in the intervention groups, and from 0.894 (T1) to 0.907 (T4) in the control group.

In addition to the abovementioned psychological variables, the questionnaire included items on the socio-demographic characteristics of the participants (i.e., gender, age, educational level, employment status) and other questions regarding their participation in the training sessions, such as their evaluation of the training (on a 5-point Likert-type scale where 1 = Unsatisfactory and 5 = Excellent), their level of commitment to the practice of the proposed exercises (on a scale from 0 to 100%), and the number of days per week that they performed formal and informal practice exercises.

### Data analyses

The analyses mainly rely on the use of the General Linear Model. A series of two-factor (Treatment and Time) mixed ANOVA 3 × 4, with a between-groups factor (Treatment: MSC, MBSR, and CG) and one factor involving repeated measures—Time: pre- (T1), post-8 week training (T2), 6-month continued practice follow-up (T3), and 12-month continued practice follow-up (T4)—were conducted, with psychological variables as the outcome variables. The potential confounding effects of sex and age were controlled for in the analyses.

This procedure mainly focused on the analyses of the Treatment × Time interaction, which allows establishing whether the different conditions, i.e., MSC, MBSR, and CG, have changed differently across time. Significant interactions were further analysed by means of planned 2 × 2 contrasts (MBSR vs. GC, MSC vs. GC and MBSR vs. MSC).

The sensitivity analyses carried out by means of the programme G*Power indicated that for the proposed design (3 conditions and 4 moments of measurement) medium effect sizes for between-factors (Cohen’s *f* = 0.24; *r* = 0.23), within-factors (Cohen’s *f* = 0.25; *r* = 0.24), and interactions (Cohen’s *f* = 0.28; *r* = 0.27) could be detected, assuming a *α* = 0.05 and a statistical power of 80%.

Intention-To-Treat (ITT) with last-observation-carried-forward (LOCF) methodology and Per-Protocol (PP) strategies were used in data analyses. The results obtained using ITT and PP analyses were compared as suggested by the Statistical Principles for Clinical Trials of the ICH E9 harmonised guidelines (European Medicines Agency, Sept. 1998, CPMP/ICH/363/96).

Analyses were carried out using the statistical software package SPSS 28 (IBM Corp., Armonk, NY, USA).

## Results

At pre-intervention measurement, ANOVA revealed differences in the mean age of the three groups (Brown-Forsythe *F*_2,155.65_ = 16.214, *p* = 000), with post-hoc contrasts (Games-Howell) indicating that CG differed significantly from the two experimental conditions (MSC and MBSR). The Chi-square test did not, however, reveal any differences in the gender composition of the three groups (Chi-square = 2.578, *gl* = 2, *p* = 0.276). As previously commented, age and gender were controlled for in ANOVA-based analyses. There were no significant differences in the number of sessions attended by participants in the MSC (*Mean* = 8.33, *Sd* = 0.88) and MBSR (*Mean* = 8.42, *Sd* = 0.65) groups during the standard 8-week training period (*t* = 0.610, *gl* = 105, *p* = 0.543).

In both MSC and MBSR experimental conditions, the evaluation of the training received was high. However, the participants rated MSC training more positively than MBSR at T2 (MSC: mean = 4.81, *Sd* = 0.39; MBSR: mean = 4.42, *Sd* = 0.62; *t*_99.54_ = 3.93, *p* < 0.000) and at T4 (MSC: mean = 4. 80, *Sd* = 0.40; MBSR: mean = 4.44, *Sd* = 0.87; *t*_63.25_ = 2.51, *p* = 0.015), but not at T3 (MSC: mean = 4.66, *Sd* = 0.61; MBSR: mean = 4.40, *Sd* = 0.88; *t*_87.27_ = 1.68, *p* = 0.097).

With respect to the level of personal engagement with the training, no differences were observed between the two experimental conditions at any of the measurement moments, T2 (MSC: mean = 76.40, *Sd* = 15.20; MBSR: mean = 78. 12, *Sd* = 13.16; *t*_105_ = −0.63, *p* = 0.531), T3 (MSC: mean = 72.73, *Sd* = 14.96; MBSR: mean = 72.94, *Sd* = 14.99; *t*_92_ = −0.07, *p* = 0.95) and T4 (MSC: mean = 77.15, *Sd* = 13.93; MBSR: mean = 75.38, *Sd* = 14.73; *t*_84_ = 0.57, *p* = 0.57).

The levels of formal practice were higher in the MBSR group, where a greater number of days per week were invested than the MSC group at T2 (MSC: mean = 4.10, *Sd* = 1.57; MBSR: mean = 4.98, *Sd* = 1. 14; *t*_83.21_ = −3.24, *p* = 0.002), T3 (MSC: mean = 3.05, *Sd* = 1.52; MBSR: mean = 4.20, *Sd* = 1.14; *t*_92_ = −4.18, *p* < 0.001) and T4 (MSC: mean = 3.07, *Sd* = 1.49; MBSR: mean = 4.16, *Sd* = 1.41; *t*_84_ = −3.46, *p* < 0.001). There were, however, no differences between the two experimental conditions concerning the number of days per week that participants engaged in informal practice exercises at T2 (MSC: mean = 5.35, *Sd* = 1.44; MBSR: mean = 5. 64, *Sd* = 1.42; *t*_105_ = −1.04, *p* = 0.299), T3 (MSC: mean = 5.39, *Sd* = 1.26; MBSR: mean = 5.42, *Sd* = 1.44; *t*_92_ = −0.12, *p* = 0.905) and T4 (MSC: mean = 5.61, *Sd* = 1.34; MBSR: mean = 5.16, *Sd* = 1.49; *t*_84_ = 1.48, *p* = 0.143).

As shown in Tables 1, 2, participants initially reported intermediate levels on all psychological variables measured, except for anxiety and depression, where baseline levels can be considered low.

**Table 1:** Means and standard deviations for the study variables, by experimental condition and time, when ITT data analysis strategy is used.

Variable (range)	Group	T1		T2		T3		T4	
		Mean	SD	Mean	SD	Mean	SD	Mean	SD
Psychological flexibility (1–5)	MSC	3.15	0.88	3.73	0.61	3.82	0.63	3.93	0.55
	MBSR	3.02	0.82	3.54	0.71	3.62	0.70	3.69	0.76
	CG	3.27	0.75	3.34	0.76	3.40	0.71	3.44	0.82
	TOTAL	3.14	0.82	3.53	0.71	3.60	0.70	3.67	0.75
Self compassion (1–5)	MSC	2.97	0.63	3.50	0.45	3.68	0.48	3.84	0.40
	MBSR	2.91	0.69	3.21	0.66	3.32	0.68	3.40	0.73
	CG	3.04	0.54	3.07	0.62	3.11	0.59	3.13	0.57
	TOTAL	2.97	0.62	3.24	0.61	3.35	0.64	3.43	0.66
Mindfulness (1–6)	MSC	3.43	1.00	4.05	0.83	4.41	0.70	4.48	0.64
	MBSR	3.58	0.97	3.87	0.75	4.19	0.76	4.22	0.77
	CG	3.90	0.97	3.92	1.05	3.92	1.02	3.94	1.00
	TOTAL	3.65	0.99	3.94	0.88	4.16	0.86	4.20	0.84
Presence of meaning in life (1–7)	MSC	4.62	1.53	5.21	1.23	5.28	1.19	5.28	1.23
	MBSR	4.64	1.14	4.95	1.08	5.12	1.15	5.00	1.17
	CG	4.94	1.26	4.95	1.40	4.75	1.34	4.87	1.28
	TOTAL	4.73	1.30	5.02	1.23	5.04	1.24	5.03	1.23
Cognitive fusion (1–7)	MSC	4.18	1.44	3.17	1.27	2.91	1.04	2.68	0.96
	MBSR	4.33	1.49	3.56	1.34	3.08	1.36	3.26	1.37
	CG	4.00	1.33	3.89	1.46	3.86	1.36	3.82	1.39
	TOTAL	4.18	1.42	3.56	1.38	3.29	1.33	3.29	1.34
Experiential avoidance (1–7)	MSC	3.57	1.48	2.80	1.14	2.40	1.05	2.48	1.03
	MBSR	3.96	1.43	3.36	1.42	3.04	1.38	3.01	1.42
	CG	3.31	1.29	3.27	1.27	3.18	1.24	3.18	1.27
	TOTAL	3.63	1.42	3.17	1.31	2.91	1.28	2.92	1.29
Behaviour activation (0–6)	MSC	3.99	1.25	4.55	0.80	4.60	0.78	4.73	0.78
	MBSR	4.33	0.95	4.45	1.01	4.70	0.94	4.51	1.14
	CG	4.12	1.22	4.12	1.30	4.25	1.11	4.25	1.02
	TOTAL	4.16	1.13	4.37	1.07	4.52	0.97	4.48	1.02
Anxiety (0–21)	MSC	9.17	4.34	6.67	2.91	6.27	2.61	5.69	2.75
	MBSR	8.42	3.70	6.95	3.27	6.49	2.97	6.38	3.41
	CG	7.47	3.91	7.81	4.07	7.51	4.03	7.21	4.05
	TOTAL	8.31	3.99	7.16	3.48	6.77	3.30	6.46	3.51
Depression (0–21)	MSC	4.98	3.63	2.85	2.45	3.00	2.51	2.42	2.39
	MBSR	4.69	3.49	3.12	3.05	2.86	2.63	3.00	2.93
	CG	4.26	3.23	4.05	3.15	4.00	3.41	4.09	3.74
	TOTAL	4.63	3.44	3.36	2.95	3.28	2.91	3.20	3.15
Perceived stress (0–4)	MSC	1.79	0.73	1.38	0.51	1.39	0.54	1.33	0.55
	MBSR	1.92	0.64	1.60	0.68	1.49	0.62	1.45	0.66
	CG	1.87	0.64	1.80	0.57	1.77	0.59	1.72	0.61
	TOTAL	1.87	0.66	1.61	0.62	1.55	0.60	1.51	0.63
Positive affect (1–5)	MSC	3.28	0.79	3.72	0.69	3.71	0.61	3.83	0.61
	MBSR	3.46	0.64	3.76	0.63	3.73	0.62	3.78	0.60
	CG	3.52	0.72	3.58	0.68	3.52	0.62	3.59	0.65
	TOTAL	3.43	0.71	3.69	0.67	3.65	0.62	3.73	0.62
Negative affect (1–5)	MSC	2.52	0.91	2.13	0.74	2.04	0.69	1.92	0.65
	MBSR	2.72	0.84	2.21	0.78	2.06	0.77	2.05	0.84
	CG	2.46	0.80	2.42	0.80	2.41	0.89	2.34	0.86
	TOTAL	2.58	0.85	2.26	0.78	2.17	0.80	2.11	0.81
Happiness (1–7)	MSC	4.52	1.25	4.88	1.03	5.03	1.04	5.19	1.03
	MBSR	4.53	1.27	4.81	1.19	5.00	1.16	5.00	1.18
	CG	4.70	1.16	4.83	1.16	4.82	1.15	4.81	1.16
	TOTAL	4.58	1.22	4.83	1.13	4.95	1.12	4.99	1.13
Satisfaction with life (1–7)	MSC	4.34	1.32	4.84	0.93	4.94	0.92	4.99	0.92
	MBSR	4.29	1.31	4.59	1.17	4.81	1.10	4.84	1.12
	CG	4.43	1.34	4.57	1.32	4.57	1.22	4.65	1.22
	TOTAL	4.35	1.32	4.66	1.16	4.76	1.10	4.82	1.11

MSC, *n* = 48; MBSR, *n* = 65; CG, *n* = 57; Total sample, *N* = 170.

**Table 2:** Means and standard deviations for the study variables, by experimental condition and time, when PP data analysis strategy is used.

Variable (range)	Group	T1		T2		T3		T4	
		Mean	SD	Mean	SD	Mean	SD	Mean	SD
Psychological flexibility (1–5)	MSC	3.24	0.83	3.80	0.56	3.84	0.66	3.97	0.57
	MBSR	2.99	0.84	3.60	0.62	3.71	0.60	3.87	0.64
	CG	3.26	0.72	3.31	0.75	3.38	0.63	3.43	0.77
	TOTAL	3.16	0.81	3.58	0.67	3.66	0.65	3.78	0.69
Self compassion (1–5)	MSC	2.98	0.57	3.48	0.45	3.68	0.52	3.87	0.43
	MBSR	2.91	0.63	3.29	0.51	3.46	0.50	3.57	0.52
	CG	3.06	0.54	3.12	0.64	3.16	0.60	3.21	0.57
	TOTAL	2.98	0.58	3.30	0.55	3.45	0.57	3.56	0.57
Mindfulness (1–6)	MSC	3.41	1.08	4.01	0.87	4.42	0.74	4.49	0.68
	MBSR	3.47	0.98	3.88	0.68	4.31	0.55	4.41	0.58
	CG	4.04	0.90	4.06	1.07	3.98	1.05	4.05	1.02
	TOTAL	3.62	1.02	3.98	0.87	4.25	0.80	4.33	0.78
Presence of meaning in life (1–7)	MSC	4.75	1.51	5.23	1.24	5.32	1.20	5.31	1.23
	MBSR	4.57	1.08	5.04	0.91	5.37	1.02	5.28	1.00
	CG	5.03	1.30	4.94	1.44	4.78	1.23	4.94	1.20
	TOTAL	4.76	1.30	5.08	1.20	5.18	1.17	5.19	1.14
Cognitive fusion (1–7)	MSC	4.12	1.42	3.17	1.31	2.89	1.08	2.66	1.03
	MBSR	4.21	1.52	3.19	1.14	2.66	1.02	2.91	1.21
	CG	3.67	1.26	3.55	1.32	3.61	1.30	3.47	1.24
	TOTAL	4.02	1.42	3.29	1.25	3.02	1.19	2.99	1.19
Experiential avoidance (1–7)	MSC	3.50	1.41	2.82	1.15	2.40	1.14	2.50	1.11
	MBSR	3.74	1.37	3.01	1.22	2.64	1.10	2.56	1.12
	CG	3.17	1.28	3.09	1.27	3.09	1.20	3.01	1.22
	TOTAL	3.49	1.37	2.97	1.21	2.69	1.17	2.67	1.16
Behaviour activation (0–6)	MSC	4.02	1.25	4.53	0.85	4.59	0.84	4.73	0.81
	MBSR	4.39	0.93	4.62	0.82	4.89	0.72	4.70	0.92
	CG	4.11	1.30	4.15	1.42	4.22	1.05	4.29	0.89
	TOTAL	4.18	1.16	4.45	1.05	4.59	0.90	4.59	0.89
Anxiety (0–21)	MSC	8.75	3.90	6.83	2.66	6.38	2.62	5.78	2.84
	MBSR	8.07	3.73	6.07	2.95	5.60	2.22	5.44	2.87
	CG	6.77	3.90	7.29	3.72	7.37	4.19	6.80	3.86
	TOTAL	7.92	3.89	6.69	3.12	6.39	3.11	5.96	3.21
Depression (0–21)	MSC	4.70	3.54	2.85	2.34	3.05	2.62	2.38	2.47
	MBSR	4.60	3.26	2.33	2.13	2.05	1.85	2.21	2.30
	CG	4.06	3.14	3.86	3.33	3.63	3.14	3.63	3.48
	TOTAL	4.47	3.31	2.96	2.66	2.86	2.61	2.69	2.80
Perceived stress (0–4)	MSC	1.75	0.70	1.35	0.48	1.36	0.56	1.29	0.55
	MBSR	1.85	0.64	1.45	0.59	1.33	0.48	1.29	0.59
	CG	1.85	0.69	1.70	0.56	1.75	0.59	1.65	0.61
	TOTAL	1.82	0.67	1.49	0.56	1.46	0.57	1.40	0.60
Positive affect (1–5)	MSC	3.31	0.81	3.75	0.70	3.73	0.61	3.86	0.61
	MBSR	3.45	0.60	3.88	0.51	3.83	0.49	3.93	0.45
	CG	3.44	0.74	3.57	0.68	3.44	0.59	3.56	0.56
	TOTAL	3.40	0.72	3.75	0.64	3.68	0.58	3.80	0.56
Negative affect (1–5)	MSC	2.47	0.81	2.13	0.73	2.05	0.70	1.94	0.66
	MBSR	2.66	0.78	2.06	0.69	1.86	0.63	1.86	0.71
	CG	2.40	0.80	2.35	0.68	2.43	0.90	2.30	0.84
	TOTAL	2.52	0.80	2.17	0.70	2.09	0.77	2.02	0.75
Happiness (1–7)	MSC	4.56	1.14	4.93	0.88	5.06	0.97	5.24	0.95
	MBSR	4.63	1.23	4.96	0.94	5.26	0.93	5.31	0.82
	CG	4.64	1.26	4.92	1.27	4.82	1.21	4.87	1.22
	TOTAL	4.61	1.20	4.94	1.02	5.06	1.04	5.16	1.01
Satisfaction with life (1–7)	MSC	4.45	1.24	4.87	0.92	4.98	0.93	5.04	0.93
	MBSR	4.37	1.22	4.77	0.99	5.07	0.90	5.10	0.96
	CG	4.53	1.37	4.69	1.45	4.71	1.34	4.89	1.26
	TOTAL	4.44	1.26	4.78	1.12	4.93	1.06	5.02	1.04

MSC, *n* = 40; MBSR, *n* = 43; CG, *n* = 35; Total (PP) sample, *N* = 118.

### Intention to treat (ITT) analysis

The 3 × 4 mixed ANOVA found significant interaction effects for all variables analysed (Table 3), indicating that the three groups have evolved differently across the four measurement points.

**Table 3:** 3 × 4 Mixed ANOVA between-groups, within-subject, and interaction effects for Intention to Treat (ITT) data analysis strategy.

Variable	Between-groups (Treatment)					Within-subject (Time)					Interaction (Treatment × Time)				
	*F*	*gl1*	*gl2*	*p*	η^2^_P_	*F*	*gl1*	*gl2*	*p*	η^2^_P_	*F*	*gl1*	*gl2*	*p*	η^2^_P_
Psychological Flexibility	2.650	2	165	0.074	0.031	7.929	2.405	396.798	0.000	0.046	6.533	4.810	396.798	0.000	0.073
Self compassion	7.182	2	165	0.001	0.080	14.554	2.181	359.924	0.000	0.081	15.944	4.363	359.924	0.000	0.162
Mindfulness	0.701	2	165	0.497	0.008	12.773	2.334	385.140	0.000	0.072	12.034	4.668	385.140	0.000	0.127
Presence of meaning in life	0.476	2	165	0.622	0.006	0.0708	2.437	402.024	0.519	0.004	4.082	4.873	402.024	0.001	0.047
Cognitive fusion	2.557	2	165	0.081	0.030	14.508	2.563	422.833	0.000	0.081	10.947	5.125	422.833	0.000	0.117
Experiential avoidance	3.046	2	165	0.050	0.036	7.527	2.293	378.301	0.000	0.044	7.167	4.585	378.301	0.000	0.080
Behaviour activation	1.241	2	165	0.292	0.015	8.217	2.463	406.347	0.000	0.047	3.122	4.925	406.347	0.009	0.036
Anxiety	0.184	2	165	0.222	0.009	4.875	2.558	422.100	0.004	0.029	6.627	5.116	422.100	0.000	0.074
Depression	0.846	2	165	0.431	0.010	4.739	2.300	379.424	0.007	0.028	4.670	4.599	379.424	0.000	0.054
Perceived stress	2.558	2	165	0.081	0.030	7.892	2.565	423.254	0.000	0.046	4.196	5.130	423.254	0.000	0.048
Positive affect	0.580	2	165	0.561	0.007	4.349	2.440	402.619	0.009	0.026	4.010	4.880	402.619	0.002	0.046
Negative affect	1.037	2	165	0.357	0.012	7.510	2.518	415.515	0.000	0.044	6.225	5.037	415.515	0.000	0.070
Happiness	0.236	2	165	0.790	0.003	2.624	2.477	408.640	0.061	0.016	2.524	4.953	408.640	0.029	0.030
Satisfaction with life	0.466	2	165	0.628	0.006	7.394	2.292	378.129	0.000	0.043	2.946	4.583	378.129	0.015	0.034

The planned contrasts comparing the MSC group with the CG revealed that the 8-week intervention produced significant effects from T1 to T2 on all variables except happiness. Interaction effects were also observed when comparing the evolution of the MSC group and the CG from T1 to T3 and from T1 to T4. For these comparisons, the happiness variable also shows significant interactions, indicating a different evolution of the MSC group and the CG from T1 to T3 and from T1 to T4 (Table 4).

**Table 4:** Planned contrasts (Method: simple, reference category T1) for Treatment × Time interaction effects found in 3 × 4 mixed ANOVA comparing MSC vs. CG using Intention to Treat (ITT) data analysis strategy.

Variable	T2 vs. T1			T3 vs. T1			T4 vs. T1		
	*F(1,101)*	*p*	η^2^_P_	*F(1,101)*	*p*	η^2^_P_	*F(1,101)*	*p*	η^2^_P_
Psychological flexibility	16.069	0.000	0.137	15.927	0.000	0.136	17.571	0.000	0.148
Self compassion	39.009	0.000	0.279	43.866	0.000	0.303	63.484	0.000	0.386
Mindfulness	19.438	0.000	0.161	44.696	0.000	0.307	35.437	0.000	0.260
Presence of meaning in life	7.967	0.006	0.073	13.918	0.000	0.121	11.551	0.000	0.103
Cognitive fusion	24.481	0.000	0.195	27.605	0.000	0.215	39.558	0.000	0.281
Experiential avoidance	25.412	0.000	0.201	25.581	0.000	0.202	27.999	0.000	0.217
Behaviour activation	5.161	0.025	0.049	5.275	0.024	0.050	10.769	0.001	0.096
Anxiety	19.785	0.000	0.164	17.511	0.000	0.148	19.716	0.000	0.163
Depression	15.359	0.000	0.132	10.266	0.002	0.092	17.645	0.000	0.149
Perceived stress	12.173	0.000	0.108	11.459	0.001	0.102	8.028	0.006	0.074
Positive affect	13.154	0.000	0.115	13.967	0.000	0.121	12.049	0.000	0.107
Negative affect	11.465	0.001	0.102	10.360	0.002	0.093	14.848	0.000	0.128
Happiness	3.237	0.075	0.031	4.879	0.029	0.046	10.058	0.002	0.091
Satisfaction with life	8.692	0.004	0.079	8.888	0.004	0.081	7.062	0.009	0.065

The planned contrasts showed different results for the comparison between the MBSR and CG group. In this case, the 8-week programme had effects on the variables psychological flexibility, self-compassion, cognitive fusion, experiential avoidance, anxiety, depression, perceived stress, and positive and negative affect. Furthermore, significant interaction effects are maintained when comparing the evolution of MBSR and CG from T1 to T3 and from T1 to T4. However, no interaction effects are observed that support a different evolution of the MBSR and CG group on behaviour activation, happiness and satisfaction with life, in any of the comparisons with baseline. Surprisingly, the 8-week programme does not seem to have had significant effects on the variables mindfulness and presence of meaning in life. However, concerning mindfulness, significant treatment × time interaction effects were observed when contrasting the evolution of the groups from T1 to T3 and from T1 to T4. As regards the presence of meaning in life the results only indicated a different evolution of MBSR and CG when comparing T3 with baseline, but not when comparing T1 and T2 or when comparing T1 and T4 (Table 5).

**Table 5:** Planned contrasts (Method: simple, reference category T1) for Treatment × Time interaction effects found in 3 × 4 mixed ANOVA comparing MBSR vs. CG using Intention to Treatment data analysis strategy.

Variable	T2 vs. T1			T3 vs. T1			T4 vs. T1		
	*F(1, 118)*	*p*	η^2^_P_	*F(1, 118)*	*p*	η^2^_P_	*F(1, 118)*	*p*	η^2^_P_
Psychological flexibility	11.377	0.001	0.088	15.362	0.000	0.115	10.786	0.001	0.084
Self compassion	7.099	0.009	0.057	9.841	0.002	0.077	11.277	0.001	0.087
Mindfulness	3.448	0.066	0.028	15.884	0.000	0.119	11.285	0.001	0.087
Presence of meaning in life	0.772	0.381	0.006	6.826	0.010	0.055	3.013	0.085	0.025
Cognitive fusion	9.647	0.002	0.076	20.661	0.000	0.149	14.319	0.000	0.108
Experiential avoidance	8.696	0.004	0.069	8.319	0.005	0.066	10.588	0.001	0.082
Behaviour activation	0.016	0.899	0.000	1.716	0.193	0.014	0.437	0.510	0.004
Anxiety	8.397	0.004	0.066	6.995	0.009	0.056	4.060	0.046	0.033
Depression	6.629	0.011	0.053	5.337	0.023	0.043	4.509	0.036	0.037
Perceived stress	6.803	0.010	0.055	9.169	0.003	0.072	6.989	0.009	0.056
Positive affect	4.270	0.041	0.035	4.048	0.046	0.033	1.343	0.249	0.011
Negative affect	12.502	0.000	0.096	14.089	0.000	0.107	12.281	0.000	0.094
Happiness	0.357	0.551	0.003	2.215	0.139	0.018	3.880	0.051	0.032
Satisfaction with life	1.542	0.217	0.013	3.372	0.069	0.028	1.876	0.173	0.016

Overall, the behaviour of the MSC and MBSR groups was quite similar, and no significant treatment × time interaction effects were found for the variables analysed, except for the self-compassion variable. The evolution of self-compassion levels in the MSC group was different from the MBSR group already from the pre- and post-intervention comparison, and also in the T3 and T4 comparisons with baseline. Participants in the MSC group increased their levels of self-compassion more intensely than members of the MBSR group. In addition, differences in the evolution of both treatment groups were observed in mindfulness skills (just in the T1 vs. T2 comparison) and in the behaviour activation (just in the T1 vs. T4 comparison). In both cases, these variables would have experienced a more intense increase in the MSC group (Table 6).

**Table 6:** Planned contrasts (Method: simple, reference category T1) for Treatment × Time interaction effects found in 3 × 4 mixed ANOVA comparing MSC vs. MBSR using Intention to Treatment data analysis strategy.

Variable	T2 vs. T1			T3 vs. T1			T4 vs. T1		
	*F(1, 109)*	*p*	η^2^_P_	*F(1, 109)*	*p*	η^2^_P_	*F(1, 109)*	*p*	η^2^_P_
Psychological flexibility	0.014	0.907	0.000	0.033	0.856	0.000	0.146	0.703	0.001
Self compassion	4.128	0.045	0.036	5.511	0.021	0.048	8.268	0.005	0.071
Mindfulness	3.987	0.048	0.035	3.466	0.065	0.031	3.876	0.052	0.034
Presence of meaning in life	2.191	0.142	0.020	0.782	0.379	0.007	1.629	0.205	0.015
Cognitive fusion	0.366	0.547	0.003	0.334	0.565	0.003	1.362	0.246	0.012
Experiential avoidance	0.321	0.572	0.003	0.560	0.456	0.005	0.094	0.759	0.001
Behaviour activation	3.381	0.069	0.030	0.452	0.503	0.004	4.445	0.037	0.039
Anxiety	1.684	0.197	0.015	1.161	0.284	0.011	3.906	0.051	0.035
Depression	0.508	0.478	0.005	0.015	0.904	0.000	1.426	0.235	0.013
Perceived stress	0.095	0.758	0.001	0.801	0.373	0.007	0.365	0.547	0.003
Positive affect	0.849	0.359	0.008	0.760	0.385	0.007	2.468	0.119	0.022
Negative affect	2.363	0.127	0.021	3.176	0.078	0.028	0.743	0.390	0.007
Happiness	0.049	0.826	0.000	0.000	0.992	0.000	0.906	0.343	0.008
Satisfaction with life	0.495	0.483	0.005	0.001	0.982	0.000	0.004	0.949	0.000

As shown in Table 7, when using an ITT analysis strategy, the mean effect sizes are always larger when contrasting the MSC group vs. the CG, compared to the contrasts involving the MBSR group vs. CG. For contrasts involving MSC group vs. CG, medium-large effects (self-compassion, mindfulness, cognitive fusion, experiential avoidance) and medium effects (psychological flexibility, presence of meaning, behaviour activation, anxiety, depression, perceived stress, positive affect, negative affect, happiness, satisfaction with life) were obtained. However, the contrasts comparing the evolution of the MBSR and CG groups, yielded average effects of medium size (psychological flexibility, self-compassion, mindfulness, cognitive fusion, experiential avoidance, anxiety, depression, perceived stress, negative affect), medium-small (presence of meaning, happiness, satisfaction with life) and small size (behaviour activation, positive affect).

**Table 7:** Effect sizes (*r*) for the Treatment × Time interaction effects, using PP and ITT strategies.

Variable	Comparison	Per protocol analysis							Intention to treat analysis						
		T2 vs. T1	T3 vs. T1	T4 vs. T1	MEAN ES	SE	95% CI BCA LL	95% CI BCA UL	T2 vs. T1	T3 vs. T1	T4 vs. T1	MEAN ES	SE	95% CI BCA LL	95% CI BCA UL
Psychological flexibility	MSC vs. CG	0.353	0.325	0.351	0.343	0.008	0.325	0.353	0.370	0.369	0.385	0.375	0.004	0.370	0.380
	MBSR vs. CG	0.342	0.437	0.415	0.398	0.024	0.366	0.430	0.297	0.339	0.289	0.308	0.013	0.294	0.325
	MSC vs. MBSR	0.094	0.126	0.164	0.128	0.016	0.094	0.164	0.011	0.017	0.037	0.022	0.006	0.015	0.028
Self compassion	MSC vs. CG	0.514	0.534	0.603	0.550	0.022	0.514	0.603	0.528	0.550	0.621	0.566	0.022	0.543	0.590
	MBSR vs. CG	0.314	0.380	0.402	0.365	0.022	0.314	0.402	0.238	0.277	0.295	0.270	0.013	0.251	0.289
	MSC vs. MBSR	0.059	0.035	0.111	0.068	0.018	0.035	0.111	0.191	0.219	0.266	0.225	0.017	0.210	0.241
Mindfulness	MSC vs. CG	0.414	0.628	0.544	0.529	0.052	0.457	0.600	0.402	0.554	0.510	0.489	0.037	0.438	0.539
	MBSR vs. CG	0.267	0.503	0.461	0.410	0.061	0.332	0.489	0.168	0.344	0.295	0.269	0.043	0.210	0.328
	MSC vs. MBSR	0.063	0.017	0.004	0.028	0.015	0.013	0.043	0.188	0.176	0.185	0.183	0.003	0.176	0.188
Presence of meaning in life	MSC vs. CG	0.289	0.373	0.309	0.324	0.021	0.302	0.345	0.270	0.348	0.320	0.313	0.019	0.287	0.339
	MBSR vs. CG	0.236	0.438	0.372	0.349	0.050	0.281	0.416	0.081	0.234	0.158	0.158	0.036	0.107	0.209
	MSC vs. MBSR	0.008	0.124	0.099	0.077	0.029	0.038	0.116	0.140	0.084	0.121	0.115	0.014	0.084	0.140
Cognitive fusion	MSC vs. CG	0.424	0.484	0.533	0.480	0.026	0.424	0.533	0.442	0.463	0.531	0.479	0.021	0.456	0.501
	MBSR vs. CG	0.337	0.504	0.382	0.408	0.042	0.367	0.448	0.275	0.386	0.329	0.330	0.026	0.311	0.349
	MSC vs. MBSR	0.088	0.256	0.033	0.126	0.055	0.070	0.182	0.058	0.055	0.111	0.075	0.015	0.057	0.092
Experiential avoidance	MSC vs. CG	0.369	0.428	0.414	0.404	0.015	0.384	0.423	0.448	0.450	0.466	0.455	0.005	0.449	0.460
	MBSR vs. CG	0.291	0.300	0.345	0.312	0.013	0.291	0.345	0.262	0.257	0.287	0.269	0.007	0.260	0.277
	MSC vs. MBSR	0.044	0.048	0.107	0.066	0.016	0.044	0.107	0.054	0.071	0.029	0.051	0.010	0.037	0.071
Behaviour activation	MSC vs. CG	0.245	0.230	0.324	0.266	0.024	0.230	0.324	0.220	0.223	0.310	0.251	0.024	0.222	0.280
	MBSR vs. CG	0.045	0.195	0.131	0.124	0.036	0.074	0.174	0.012	0.120	0.061	0.064	0.026	0.045	0.084
	MSC vs. MBSR	0.096	0.044	0.095	0.078	0.014	0.044	0.096	0.173	0.064	0.198	0.145	0.034	0.064	0.198
Anxiety	MSC vs. CG	0.367	0.376	0.396	0.380	0.007	0.367	0.396	0.405	0.384	0.404	0.398	0.006	0.384	0.405
	MBSR vs. CG	0.343	0.340	0.293	0.325	0.013	0.309	0.342	0.258	0.237	0.182	0.226	0.018	0.182	0.258
	MSC vs. MBSR	0.078	0.097	0.021	0.065	0.018	0.040	0.091	0.123	0.103	0.186	0.137	0.020	0.116	0.158
Depression	MSC vs. CG	0.287	0.208	0.300	0.265	0.024	0.208	0.300	0.363	0.304	0.386	0.351	0.020	0.304	0.378
	MBSR vs. CG	0.356	0.305	0.264	0.308	0.022	0.291	0.325	0.231	0.208	0.192	0.210	0.009	0.197	0.223
	MSC vs. MBSR	0.158	0.259	0.078	0.165	0.043	0.131	0.199	0.068	0.012	0.114	0.065	0.024	0.031	0.099
Perceived stress	MSC vs. CG	0.260	0.300	0.229	0.263	0.017	0.250	0.276	0.328	0.319	0.271	0.306	0.014	0.271	0.328
	MBSR vs. CG	0.224	0.336	0.265	0.275	0.027	0.251	0.299	0.233	0.269	0.236	0.246	0.010	0.235	0.257
	MSC vs. MBSR	0.058	0.213	0.181	0.151	0.039	0.099	0.202	0.030	0.085	0.058	0.058	0.013	0.039	0.076
Positive affect	MSC vs. CG	0.308	0.328	0.295	0.310	0.008	0.304	0.317	0.339	0.349	0.326	0.338	0.005	0.330	0.346
	MBSR vs. CG	0.259	0.289	0.210	0.253	0.019	0.226	0.279	0.187	0.182	0.106	0.158	0.021	0.106	0.187
	MSC vs. MBSR	0.085	0.092	0.000	0.059	0.024	0.028	0.090	0.088	0.083	0.149	0.107	0.017	0.086	0.127
Negative affect	MSC vs. CG	0.279	0.299	0.323	0.300	0.010	0.279	0.323	0.319	0.305	0.358	0.327	0.013	0.314	0.340
	MBSR vs. CG	0.364	0.447	0.369	0.393	0.022	0.367	0.419	0.310	0.327	0.307	0.315	0.005	0.309	0.321
	MSC vs. MBSR	0.253	0.355	0.257	0.288	0.028	0.256	0.321	0.146	0.168	0.082	0.132	0.021	0.082	0.168
Happiness	MSC vs. CG	0.097	0.204	0.268	0.190	0.041	0.097	0.268	0.176	0.215	0.301	0.231	0.029	0.202	0.259
	MBSR vs. CG	0.000	0.214	0.279	0.164	0.069	0.000	0.279	0.055	0.136	0.178	0.123	0.028	0.082	0.164
	MSC vs. MBSR	0.022	0.112	0.041	0.058	0.023	0.035	0.082	0.021	0.000	0.091	0.037	0.022	0.014	0.061
Satisfaction with life	MSC vs. CG	0.218	0.222	0.154	0.198	0.018	0.175	0.221	0.281	0.284	0.256	0.274	0.007	0.256	0.284
	MBSR vs. CG	0.194	0.281	0.195	0.223	0.024	0.195	0.252	0.114	0.167	0.125	0.135	0.013	0.121	0.149
	MSC vs. MBSR	0.080	0.201	0.153	0.145	0.029	0.104	0.185	0.067	0.003	0.006	0.025	0.017	0.003	0.047

### Per protocol (PP) analysis

Results from 3 × 4 mixed ANOVA revealed significant treatment-by-time interaction effects for all variables analysed, with the exception of behaviour activation, which nevertheless reached a marginally significant level (*p* = 0.051) (Table 8). These results suggest that the three groups have evolved differently across the different measurement times.

**Table 8:** 3 × 4 Mixed ANOVA between-groups, within-subject, and interaction effects using PP strategy.

Variable	Between-groups (Treatment)					Within-subject (Time)					Interaction (Treatment × Time)				
	*F*	*gl1*	*gl2*	*p*	η^2^_P_	*F*	*gl1*	*gl2*	*p*	η^2^_P_	*F*	*gl1*	*gl2*	*p*	η^2^_P_
Psychological flexibility	2.993	2	113	0.054	0.050	6.423	2.552	288.359	0.000	0.054	5.218	5.104	288.359	0.000	0.085
Self compassion	5.048	2	113	0.008	0.082	15.982	2.312	261.262	0.000	0.124	11.108	4.624	261.262	0.000	0.164
Mindfulness	0.178	2	113	0.837	0.003	13.065	2.590	292.700	0.000	0.104	12.212	5.181	292.700	0.000	0.178
Presence of meaning in life	0.204	2	113	0.816	0.004	3.307	2.616	295.658	0.026	0.028	5.082	5.233	295.658	0.000	0.083
Cognitive fusion	0.442	2	113	0.644	0.008	11.121	2.700	305.154	0.000	0.090	8.793	5.401	305.154	0.000	0.135
Experiential avoidance	0.552	2	113	0.578	0.010	4.996	2.316	261.759	0.005	0.042	5.046	4.633	261.759	0.000	0.082
Behaviour activation	1.706	2	113	0.186	0.029	6.738	2.696	304.703	0.000	0.056	2.183	5.393	304.703	0.051	0.037
Anxiety	0.292	2	113	0.747	0.005	1.892	2.760	311.901	0.136	0.016	4.791	5.520	311.901	0.000	0.078
Depression	0.700	2	113	0.499	0.012	4.700	2.351	265.611	0.007	0.040	3.447	4.701	265.611	0.006	0.057
Perceived stress	1.794	2	113	0.171	0.031	6.371	2.676	302.423	0.000	0.053	2.972	5.353	302.423	0.010	0.050
Positive affect	1.884	2	113	0.157	0.032	6.002	2.422	273.700	0.001	0.050	3.153	4.844	273.700	0.009	0.053
Negative affect	0.618	2	113	0.541	0.011	4.864	2.654	299.942	0.004	0.041	6.098	5.309	299.942	0.000	0.097
Happiness	0.715	2	113	0.491	0.012	4.880	2.624	296.496	0.004	0.041	2.439	5.248	296.496	0.032	0.041
Satisfaction with life	0.039	2	113	0.962	0.001	7.740	2.450	276.829	0.000	0.064	2.308	4.900	276.829	0.046	0.039

The planned contrasts showed that the MSC and CG groups had evolved differently over time, at any of the comparisons with baseline, on the variables psychological flexibility, self-compassion, mindfulness, presence of meaning in life, cognitive fusion, experiential avoidance, anxiety, perceived stress, and positive and negative affect. In contrast, the MSC and CG groups evolved similarly on the life satisfaction variable across all comparisons with baseline. Concerning behaviour activation and depression variables, significant treatment-by-time interaction effects were observed for the T2 and T4 comparisons with baseline, but not for the T1 vs. T3 comparison. The happiness variable showed significant treatment × time interaction effects when comparing T4 to baseline, which suggests that the differences between the MSC and CG groups are different when comparing the trajectory of both groups over a longer term (1-year follow-up) (Table 9).

**Table 9:** Planned contrasts (Method: simple, reference category T1) for Treatment × Time interaction effects found in 3 × 4 mixed ANOVA comparing MSC vs. CG, PP strategy.

Variable	T2 vs. T1			T3 vs. T1			T4 vs. T1		
	*F(1, 71)*	*p*	η^2^_P_	*F(1, 71)*	*p*	η^2^_P_	*F(1, 71)*	*p*	η^2^_P_
Psychological flexibility	10.119	0.002	0.125	8.368	0.005	0.105	9.970	0.002	0.123
Self compassion	25.522	0.000	0.264	28.263	0.000	0.285	40.552	0.000	0.364
Mindfulness	14.658	0.000	0.171	46.157	0.000	0.394	29.853	0.000	0.296
Presence of meaning in life	6.494	0.013	0.084	11.476	0.001	0.139	7.474	0.008	0.095
Cognitive fusion	15.594	0.000	0.180	21.709	0.000	0.234	28.127	0.000	0.284
Experiential avoidance	11.172	0.001	0.136	15.939	0.000	0.183	14.663	0.000	0.171
Behaviour activation	4.551	0.036	0.060	3.949	0.051	0.053	8.351	0.005	0.105
Anxiety	11.072	0.001	0.135	11.704	0.001	0.142	13.244	0.001	0.157
Depression	6.393	0.014	0.083	3.203	0.078	0.043	7.007	0.010	0.090
Perceived stress	5.155	0.026	0.068	7.047	0.010	0.090	3.938	0.051	0.053
Positive affect	7.467	0.008	0.095	8.550	0.005	0.107	6.786	0.011	0.087
Negative affect	5.992	0.017	0.078	6.983	0.010	0.090	8.262	0.005	0.104
Happiness	0.678	0.413	0.009	3.098	0.083	0.042	5.514	0.022	0.072
Satisfaction with life	3.559	0.063	0.048	3.667	0.060	0.049	1.734	0.192	0.024

The MBSR and CG groups showed significantly different trajectories, in all comparisons with baseline, in the variables psychological flexibility, self-compassion, mindfulness, presence of meaning in life, cognitive fusion, experiential avoidance, anxiety, depression, and perceived stress (although for the latter variable at a marginally significant level in the T1 vs. T2 comparison, with *p* = 0.051). Significant treatment-by-time interaction effects were also obtained in the T1 vs. T4 comparison for the levels of happiness, and in the T1 vs. T3 comparison for satisfaction with life. Planned contrasts indicated that the MBSR and CG groups had similar trajectories on the behaviour activation variable in all comparisons with baseline (Table 10).

**Table 10:** Planned contrasts (Method: simple, reference category T1) for Treatment × Time interaction effects found in 3 × 4 mixed ANOVA comparing MBSR vs. CG, PP strategy.

Variable	T2 vs. T1			T3 vs. T1			T4 vs. T1		
	*F(1, 74)*	*p*	η^2^_P_	*F(1, 74)*	*p*	η^2^_P_	*F(1, 74)*	*p*	η^2^_P_
Psychological flexibility	9.820	0.002	0.117	17.445	0.000	0.191	15.387	0.000	0.172
Self compassion	8.096	0.006	0.099	12.484	0.001	0.144	14.273	0.000	0.162
Mindfulness	5.702	0.020	0.072	25.072	0.000	0.253	19.957	0.000	0.212
Presence of meaning in life	4.375	0.040	0.056	17.602	0.000	0.192	11.887	0.001	0.138
Cognitive fusion	9.471	0.003	0.113	25.154	0.000	0.230	12.635	0.001	0.146
Experiential avoidance	6.836	0.011	0.085	7.327	0.008	0.090	9.997	0.002	0.119
Behaviour activation	0.151	0.698	0.002	2.929	0.091	0.038	1.297	0.258	0.017
Anxiety	9.858	0.002	0.118	9.655	0.003	0.115	6.965	0.010	0.086
Depression	10.765	0.002	0.127	7.566	0.007	0.093	5.532	0.021	0.070
Perceived stress	3.919	0.051	0.050	9.426	0.003	0.113	5.569	0.021	0.070
Positive affect	5.339	0.024	0.067	6.769	0.011	0.084	3.419	0.068	0.044
Negative affect	11.269	0.001	0.132	18.431	0.000	0.199	11.696	0.001	0.136
Happiness	0.000	0.995	0.000	3.549	0.064	0.046	6.235	0.015	0.078
Satisfaction with life	2.885	0.094	0.038	6.355	0.014	0.079	2.926	0.091	0.038

Overall, the MSC and MBSR groups had similar trajectories in all comparisons to baseline, with some exceptions (Table 11). Participants in the MBSR group reduced their negative affect scores more strongly than participants in the MSC programme in all comparisons with baseline. Furthermore, in the comparison between T1 and T3, the MBSR group reduced their cognitive fusion and depression scores more intensely than the MSC group.

**Table 11:** Planned contrasts (Method: simple, reference category T1) for Treatment × Time interaction effects found in 3 × 4 mixed ANOVA comparing MSC vs. MBSR, PP strategy.

Variable	T2 vs. T1			T3 vs. T1			T4 vs. T1		
	*F(1, 79)*	*p*	η^2^_P_	*F(1, 79)*	*p*	η^2^_P_	*F(1, 79)*	*p*	η^2^_P_
Psychological flexibility	0.704	0.404	0.009	1.266	0.264	0.016	2.171	0.145	0.027
Self compassion	0.279	0.599	0.004	0.099	0.754	0.001	0.994	0.322	0.012
Mindfulness	0.316	0.576	0.004	0.022	0.882	0.000	0.001	0.974	0.000
Presence of meaning in life	0.005	0.946	0.000	1.240	0.269	0.015	0.783	0.379	0.010
Cognitive fusion	0.620	0.433	0.008	5.520	0.021	0.065	0.084	0.773	0.001
Experiential avoidance	0.155	0.695	0.002	0.186	0.667	0.002	0.908	0.344	0.011
Behaviour activation	0.733	0.394	0.009	0.154	0.696	0.002	0.727	0.397	0.009
Anxiety	0.486	0.488	0.006	0.753	0.388	0.009	0.035	0.852	0.000
Depression	2.030	0.158	0.025	5.676	0.020	0.067	0.489	0.486	0.006
Perceived stress	0.264	0.609	0.003	3.740	0.057	0.045	2.684	0.105	0.033
Positive affect	0.576	0.450	0.007	0.674	0.414	0.008	0.000	0.994	0.000
Negative affect	5.404	0.023	0.064	11.418	0.001	0.126	5.583	0.021	0.066
Happiness	0.037	0.848	0.000	1.004	0.319	0.013	0.134	0.715	0.002
Satisfaction with life	0.505	0.479	0.006	3.333	0.072	0.040	1.896	0.172	0.023

As shown in Table 7, the Treatment × Time interactions obtained in the planned contrasts concerning psychological flexibility, presence of meaning in life, experiential avoidance, anxiety, depression, perceived stress, and positive and negative affect, presented a medium effect size, when comparing the evolution of the MSC and MBSR groups with the CG, through the different comparisons with the baseline (T2 vs. T1; T3 vs. T1; T4 vs. T1). In the case of mindfulness and cognitive fusion, the evolution of the MSC group globally presents medium-large differences with respect to the CG, while the differences in the evolution of the MBSR group with respect to the CG can be considered medium-sized. For the satisfaction with life variable, the effect sizes obtained can be considered medium-small. We also found medium-small effect sizes for happiness, except for the comparison T1 vs. T2, where the effect sizes are small. Regarding the variable self-compassion, it is remarkable that large effect sizes were observed for the contrasts involving MSC group vs. CG, whereas the effect sizes for the contrasts involving MBSR group vs. CG were medium. The differences in the evolution of the MSC group and the CG with respect to behaviour activation can be considered medium or small, while the differences between the MBSR and CG trajectories are small.

## Discussion

To the best of our knowledge, this is the first study to compare MSC training with MBSR training which also includes a year of continuous mindfulness and/or self-compassion practice. Although the design is not strictly an RCT, as the CG was not randomised, the arm corresponding to the MSC vs. MBSR comparison did include randomisation. In this sense, this study adds to the still scarce literature comparing the MSC programme with a well-established intervention such as MBSR.

### Effectiveness of the MSC training

Our research is aligned with previous studies that highlighted the benefits of the MSC training (Yela et al., 2020a), and overall we obtained medium effect-sizes in the comparisons between MSC and CG. Concerning the efficacy of the MSC programme, our results revealed that the 8-week standard programme, compared with CG, produced changes in Psychological Flexibility, Self-Compassion, Mindfulness, Presence of Meaning in Life, Cognitive Fusion, Experiential Avoidance, Behaviour Activation, Anxiety, Depression, Perceived Stress, Positive Affect, and Negative Affect. These findings are coherent with those reported in previous research by Kirby et al. (2017) in which self-compassion-based interventions found significant changes in mindfulness, self-compassion, anxiety, psychological distress and wellbeing, with intermediate effect sizes. Similarly, Ferrari et al.’s (2019) meta-analysis found improved levels of mindfulness, self-compassion, self-criticism, anxiety and depression, with intermediate effect sizes. Finally, our results are in line with reviews in which self-compassion-focused trainings were found to significantly improved depression and stress levels with a medium effect size, and depression levels with a small effect size (Han and Kim, 2023). In a similar vein, the benefits of LKCM training on depression and anxiety have been reported by Lv et al. (2024) and Zheng et al. (2023), respectively.

Our findings concerning changes in variables related to psychological flexibility are also coherent with previous research. For instance, Yela et al. (2020b) found that mindfulness meditation produced significant changes in experiential avoidance, which is also connected to improvements in mental health.

The increased positive affect and decreased negative affect observed in our research following the 8-week MSC training are also in line with results from other studies in which the MSC programme was used (Neff and Germer, 2017; Yela et al., 2020a; Yela et al., 2022). In our study, increases in happiness levels after the 8-week MSC programme were not observed, and changes in this variable may need 12 months of continued practice in order to emerge. The 8-week MSC programme did not produce consistent changes in the life satisfaction variable, at least when a PP strategy is considered. Probably, the fact that we are working with a general population explains why the margin of change in this variable is limited. These results are similar to those reported in the review by Gu et al. (2022) in which significant improvements in this variable were only seen in studies with pre-post designs, although, as in our case, significance disappeared when only randomised controlled trials were considered. Assessments of subjective wellbeing are considered indicative of an individual’s global evaluation of his/her quality of life. As personal circumstances are generally slow to change, the judgements believed to be contingent upon these circumstances may exhibit a certain level of stability, particularly across brief timeframes (Lucas et al., 2018). In this sense, a detailed reading of the items of the scales used to assess happiness and life satisfaction shows that these instruments refer to broad appraisals of how good the respondent’s life is overall. For example, items such as ‘In most ways my life is close to my ideal’ (item 1 of SWLS) or ‘Some people are generally very happy. They enjoy life regardless of what is going on, getting the most out of everything. To what extent does this characterization describe you?’ (item 3 of SHS) may reflect with difficulty changes in a short interval of time.

### Comparing the MSC and MBSR programmes

Our research compared the MSC programme with the well-established MBSR protocol. In general, the differences in the comparisons between the MSC and MBSR groups can be considered small in terms of effect size and the MSC and MBSR groups performed similarly, which is in line with previous pre-post studies (Jiménez-Gómez et al., 2022). However, there are some nuances. Analyses using the ITT strategy show that MSC training increases levels of self-compassion more than MBSR training. This is to be expected, as self-compassion is a skill that is directly trained in MSC. The MSC training also appears to have positively influenced mindfulness at the 8-week interval and behavioural activation at the 1-year interval, compared with the MBSR group. The analyses using a PP strategy also show a generally similar trajectory for the MSC and MBSR groups. Again, however, there are some nuances. According to this analysis strategy, MBSR appears to perform better in terms of reducing negative emotions and, occasionally, depression and cognitive fusion.

Both types of training also outperformed the CG in virtually all the variables considered. Interestingly, in most cases, the most pronounced effects occur after 8 weeks, while continued practice helps to maintain (or slightly improve) the gains made after standard training. This is relevant, as participating in an 8-week programme appears to represent a turning point in the improvement of participants’ skills and psychological processes (e.g., mindfulness, self-compassion, psychological flexibility) and mental health outcomes (e.g., anxiety, stress, depression). The role of continued practice is to maintain those benefits derived from the standard trainings. However, when compared with the CG, MSC and MBSR groups sometimes behaved differently. Considering the results of the ITT strategy, it seems that MBSR training—unlike MSC—does not differ from CG in terms of behavioural activation. Even the effects of MBSR on mindfulness and meaningfulness took longer to differentiate from CG performance, compared to the MSC group. This result may be due to the presence of more drop-outs in MBSR than in MSC. However, when a PP strategy is considered, the results suggest that MBSR produces changes in all the variables analysed except behavioural activation.

In terms of the satisfaction and level of engagement with the training sessions, both trainings performed quite similar. Participants rated the MSC intervention more positively, but participants reported high levels of satisfaction in both trainings. No differences were found in the participants’ levels of engagement with the trainings despite the fact that participants in the MBSR group reported higher levels of formal practice. The relevance of these motivational variables is worth remark, as evidenced in previous studies. For example, Yela et al. (2020a) found that participants who showed a high level of adherence to the MSC programme significantly improved their scores in self-compassion, mindfulness and psychological wellbeing, in comparison with individuals with low adherence to the programme, who maintained their previous levels of self-compassion and wellbeing and just slightly increased their mindfulness skills.

### Limitations

Despite its contributions, this study has limitations that make it necessary to treat results with caution. The branch of the study that compared MSC and MBSR had a random distribution of participants. In addition, we compared the MSC group with a training for which there is already ample evidence of its beneficial effects, such as MBSR, which provides additional guarantees of the results. However, we were not able to have a fully randomised control group, which does not allow us to compare with full confidence the trainees with those who did not receive the training. This potential source of concern has been mitigated by controlling for variables such as gender and age, and by using statistical techniques that take into account the possible lack of equivalence of the groups at baseline. In this sense, the analyses performed focus on the treatment by time interaction, i.e., they compare whether the different groups had a different pattern of development over time, regardless of their initial levels. In any case, it would be interesting to include sensitivity analysis and subgroup analysis in a possible future study to confirm the robustness of the results.

As can be seen from the analyses carried out, the people in the control group were on average younger, which could affect some variables. However, in our analyses, age was one of the factors that we controlled for statistically in order to ensure that the results were not confounded by this variable. Gender was also used as a control variable in our study, as our sample was predominantly female. Studies such as Thirumaran et al. (2020) have found that mindfulness tends to increase with age and that women tend to be more mindful than men, although this gender difference was not statistically significant. In this sense, it would be interesting to explore these aspects in more detail in future studies and to analyse whether similar effects are obtained in more gender-diverse samples and in comparisons between experimental and control groups which are more similar in terms of age.

A potential limitation comes from the training being delivered by four different psychologists. For instance, each trainer may have introduced subtle differences in the way they supervised their respective groups, thus introducing an extraneous variable. However, it is important to note that the trainers adhered to the protocols and instructions of the programmes they were teaching in order to minimise the impact of therapist personal variables. This is precisely one of the benefits of using standardised protocols in research (Kendall and Frank, 2018). Furthermore, mechanisms for instructor coordination were established throughout the training period. Instructors held weekly meetings to discuss any incidents arising during the sessions and to plan the following week’s sessions and practices. These meetings helped to ensure that participants received consistent training. Another limitation is that the trainers in the MBSR group did not follow a teacher training certification pathway like the one proposed by Brown University, School of Professional Studies (2023). This may affect the orthodoxy of MBSR training, and therefore the results obtained in this group must be taken with caution, especially when comparing the MSC and MBSR groups. A conservative interpretation of the results would be to consider that the MSC group was compared to a mindfulness-based intervention with similar content and practices to MBSR.

With regard to attrition in the MBSR group, which may be another limitation, we hypothesise that one possible explanation is that this programme is more demanding in terms of formal practice requirements than the MSC programme, where practices are typically shorter. In addition, mindfulness practices need to be very well contextualised in a context where participants were primarily looking for emotional self-care training and may have expected more classical skills training or short-term outcomes. By including self-compassion practices, the MSC programme may be more in line with the participants’ initial expectations, and specific training in the self-compassion component may contribute to greater adherence among people seeking to cultivate self-care. In any case, these ideas are hypotheses that would be interesting to explore in the future.

### Implications and future directions

Both the MSC and MBSR programmes could be used preventively to reduce symptoms associated with poor mental health (depression, anxiety, stress) and increase positive indices of mental health (improved quality of life, life satisfaction, positive affect, happiness) in the general population. It is important to remember that both MSC and MBSR are not considered “psychological therapies” but rather group training programmes. Access to these programmes by the general population could play an important role in preventing the onset of mental health problems.

Our research also introduced a focus on the changes that both programmes produce in transversal processes in psychopathology framed in the field of third generation therapies, such as psychological flexibility, mindfulness, self-compassion, cognitive fusion, experiential avoidance and behavioural activation/commitment to action. This may be a first step in order to consider, as Hayes (2025) himself states, self-compassion as a radically transdiagnostic process, which favours the improvement of emotional and cognitive flexibility so important in psychotherapy. As he points out, the impact of self-compassion is “profoundly transdiagnostic.” In fact, it would make sense to start talking about interventions based on mindfulness-acceptance and self-compassion.

As mentioned above, this study is part of a larger research project analysing the effects of sustained mindfulness and self-compassion practice on wellbeing, mental health, and also on biomarkers related to cardiovascular health and immune response. Our next steps have four objectives. First, to identify possible mediating variables that may explain the beneficial effects of mindfulness and self-compassion practice observed in the present study. Second, we want to explore what characteristics of the practice, in terms of frequency and duration of sessions and type of practice (formal versus informal), might make this type of training more effective. It would also be interesting to explore facilitators and barriers to mindfulness practice. In connection with these aims, future studies might benefit from including qualitative assessments or validated self-report tools that explore practice depth and quality and perceived engagement with training. Thirdly, we will explore how the trainings and continuous practice of mindfulness/self-compassion might have affected cardiovascular health and immune response biomarkers. Fourth, we will analyse how psychological variables and biomarkers might be related.

Beyond our own research project, it would be interesting to know how the MSC programme works in a clinical population. In the present study, this training has shown that it can be an intervention with a very high level of adherence and that it is highly satisfactory for the participants. If these findings, and the psychological benefits derived from the training, are maintained in the clinical population, the MSC programme may become an optimal therapeutic tool, like other mindfulness-based programmes such as MBCT (Ferguson et al., 2021; Segal et al., 2002).

## Conclusion

The standard 8-week MSC programme has positive effects on a wide range of mental health-related variables and is comparable to another well-established programme such as MBSR. On-going practice of mindfulness and self-compassion helps to maintain the psychological benefits of the programme, particularly with regard to mental health, and even helps to produce other, longer-term benefits, such as increased levels of happiness. In this regard, the MSC programme represents an effective intervention to foster mental health and prevent depression and anxiety-related issues in the general population.

## Data Availability

The datasets presented in this article are not readily available because the data are part of a larger research programme that is still under development. Requests to access the datasets should be directed to jryelabe@upsa.es.

## Supplementary material

SUPPLEMENTARY FIGURE 1 Psychological Flexibility. Estimated marginal means are presented in Y-axis. Time is presented in X-axis: T1 = pre-training, T2 = post-training, T3 = 6-month continued practice follow-up, T4 = 1-year continued practice follow-up. Blue line = MSC; Green line = MBSR; Red line = CG.

SUPPLEMENTARY FIGURE 2 Self-compassion. Estimated marginal means are presented in Y-axis. Time is presented in X-axis: T1 = pre-training, T2 = post-training, T3 = 6-month continued practice follow-up, T4 = 1-year continued practice follow-up. Blue line = MSC; Green line = MBSR; Red line = CG.

SUPPLEMENTARY FIGURE 3 Mindfulness. Estimated marginal means are presented in Y-axis. Time is presented in X-axis: T1 = pre-training, T2 = post-training, T3 = 6-month continued practice follow-up, T4 = 1-year continued practice follow-up. Blue line = MSC; Green line = MBSR; Red line = CG.

SUPPLEMENTARY FIGURE 4 Presence of meaning in life. Estimated marginal means are presented in Y-axis. Time is presented in X-axis: T1 = pre-training, T2 = post-training, T3 = 6-month continued practice follow-up, T4 = 1-year continued practice follow-up. Blue line = MSC; Green line = MBSR; Red line = CG.

SUPPLEMENTARY FIGURE 5 Cognitive fusion. Estimated marginal means are presented in Y-axis. Time is presented in X-axis: T1 = pre-training, T2 = post-training, T3 = 6-month continued practice follow-up, T4 = 1-year continued practice follow-up. Blue line = MSC; Green line = MBSR; Red line = CG.

SUPPLEMENTARY FIGURE 6 Experiential avoidance. Estimated marginal means are presented in Y-axis. Time is presented in X-axis: T1 = pre-training, T2 = post-training, T3 = 6-month continued practice follow-up, T4 = 1-year continued practice follow-up. Blue line = MSC; Green line = MBSR; Red line = CG.

SUPPLEMENTARY FIGURE 7 Behaviour activation. Estimated marginal means are presented in Y-axis. Time is presented in X-axis: T1 = pre-training, T2 = post-training, T3 = 6-month continued practice follow-up, T4 = 1-year continued practice follow-up. Blue line = MSC; Green line = MBSR; Red line = CG.

SUPPLEMENTARY FIGURE 8 Anxiety. Estimated marginal means are presented in Y-axis. Time is presented in X-axis: T1 = pre-training, T2 = post-training, T3 = 6-month continued practice follow-up, T4 = 1-year continued practice follow-up. Blue line = MSC; Green line = MBSR; Red line = CG.

SUPPLEMENTARY FIGURE 9 Depression. Estimated marginal means are presented in Y-axis. Time is presented in X-axis: T1 = pre-training, T2 = post-training, T3 = 6-month continued practice follow-up, T4 = 1-year continued practice follow-up. Blue line = MSC; Green line = MBSR; Red line = CG.

SUPPLEMENTARY FIGURE 10 Perceived stress. Estimated marginal means are presented in Y-axis. Time is presented in X-axis: T1 = pre-training, T2 = post-training, T3 = 6-month continued practice follow-up, T4 = 1-year continued practice follow-up. Blue line = MSC; Green line = MBSR; Red line = CG.

SUPPLEMENTARY FIGURE 11 Positive affect. Estimated marginal means are presented in Y-axis. Time is presented in X-axis: T1 = pre-training, T2 = post-training, T3 = 6-month continued practice follow-up, T4 = 1-year continued practice follow-up. Blue line = MSC; Green line = MBSR; Red line = CG.

SUPPLEMENTARY FIGURE 12 Negative affect. Estimated marginal means are presented in Y-axis. Time is presented in X-axis: T1 = pre-training, T2 = post-training, T3 = 6-month continued practice follow-up, T4 = 1-year continued practice follow-up. Blue line = MSC; Green line = MBSR; Red line = CG.

SUPPLEMENTARY FIGURE 13 Happiness. Estimated marginal means are presented in Y-axis. Time is presented in X-axis: T1 = pre-training, T2 = post-training, T3 = 6-month continued practice follow-up, T4 = 1-year continued practice follow-up. Blue line = MSC; Green line = MBSR; Red line = CG.

SUPPLEMENTARY FIGURE 14 Satisfaction with life. Estimated marginal means are presented in Y-axis. Time is presented in X-axis: T1 = pre-training, T2 = post-training, T3 = 6-month continued practice follow-up, T4 = 1-year continued practice follow-up. Blue line = MSC; Green line = MBSR; Red line = CG.
