# Supplementary figures and images for: Long-term effectiveness of the Mindful Self-Compassion programme compared to a Mindfulness-Based Stress Reduction intervention: a quasi-randomised controlled trial involving regular mindfulness practice for 1 year
Source: Front Psychol. 2025 Apr 28;16:1597264. doi: 10.3389/fpsyg.2025.1597264 (PMC12066306; doi:10.3389/fpsyg.2025.1597264)

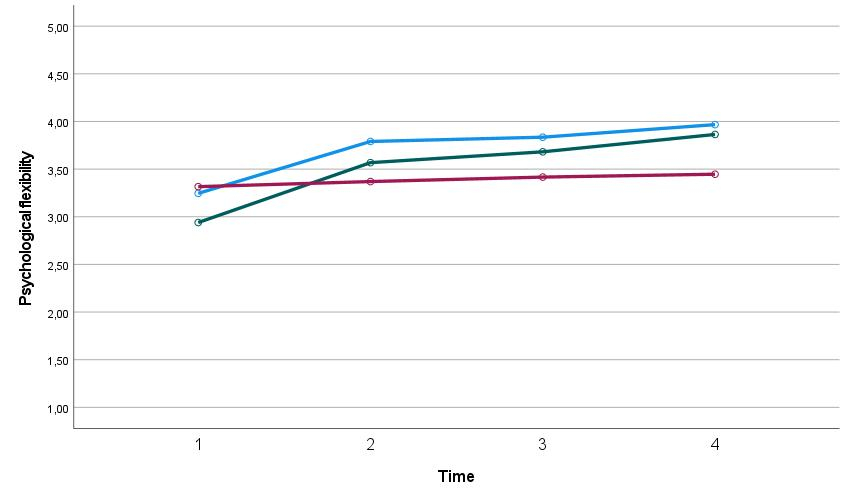

Supplement: SUPPLEMENTARY FIGURE 1 — Psychological Flexibility. Estimated marginal means are presented in Y-axis. Time is presented in X-axis: T1 = pre-training, T2 = post-training, T3 = 6-month continued practice follow-up, T4 = 1-year continued practice follow-up. Blue line = MSC; Green line = MBSR; Red line = CG. [file Image_1.JPEG]

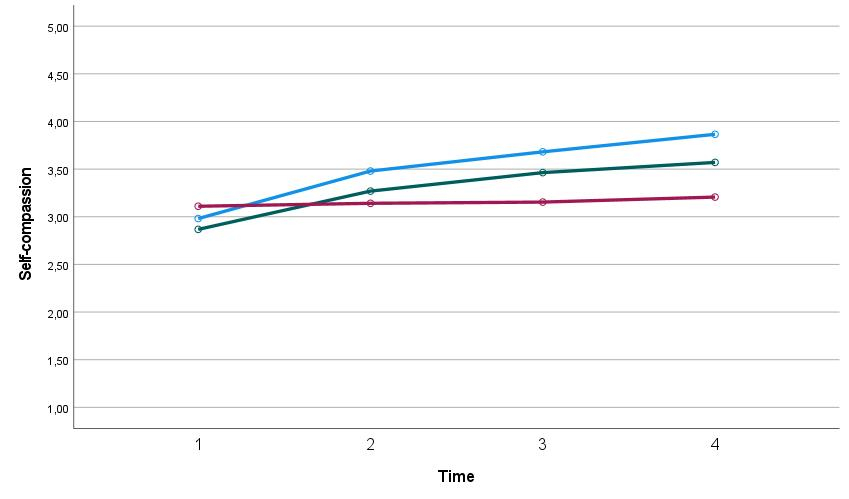

Supplement: SUPPLEMENTARY FIGURE 2 — Self-compassion. Estimated marginal means are presented in Y-axis. Time is presented in X-axis: T1 = pre-training, T2 = post-training, T3 = 6-month continued practice follow-up, T4 = 1-year continued practice follow-up. Blue line = MSC; Green line = MBSR; Red line = CG. [file Image_2.JPEG]

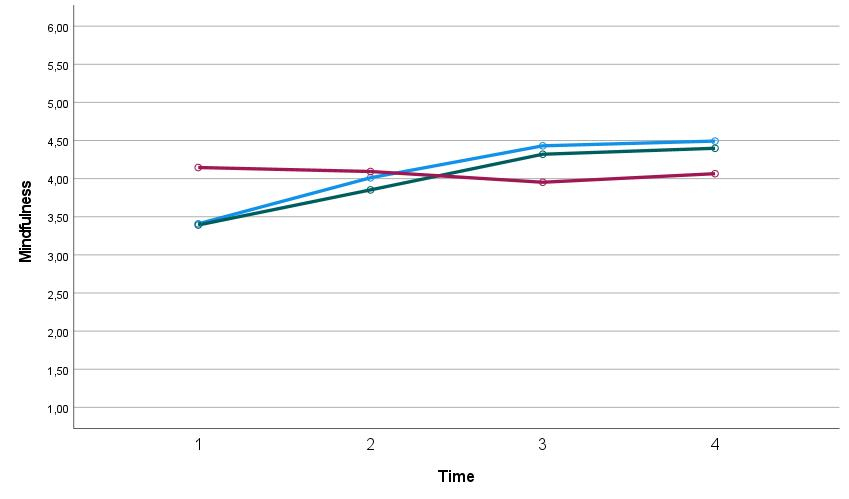

Supplement: SUPPLEMENTARY FIGURE 3 — Mindfulness. Estimated marginal means are presented in Y-axis. Time is presented in X-axis: T1 = pre-training, T2 = post-training, T3 = 6-month continued practice follow-up, T4 = 1-year continued practice follow-up. Blue line = MSC; Green line = MBSR; Red line = CG. [file Image_3.JPEG]

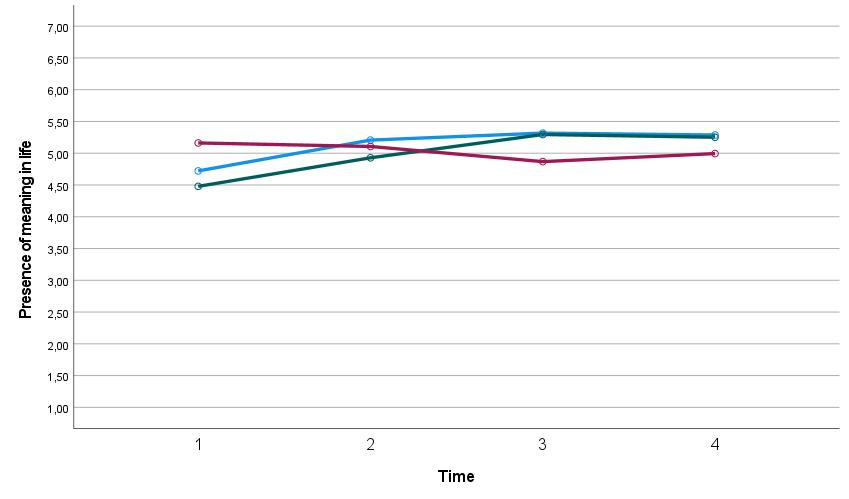

Supplement: SUPPLEMENTARY FIGURE 4 — Presence of meaning in life. Estimated marginal means are presented in Y-axis. Time is presented in X-axis: T1 = pre-training, T2 = post-training, T3 = 6-month continued practice follow-up, T4 = 1-year continued practice follow-up. Blue line = MSC; Green line = MBSR; Red line = CG. [file Image_4.JPEG]

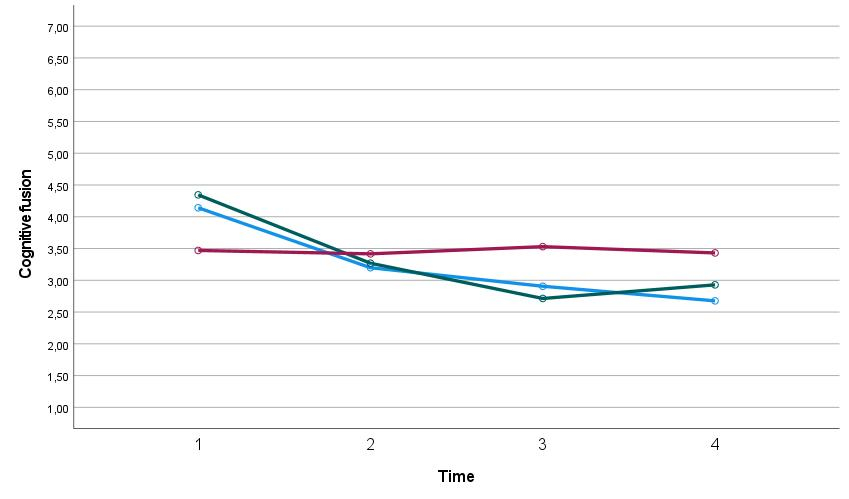

Supplement: SUPPLEMENTARY FIGURE 5 — Cognitive fusion. Estimated marginal means are presented in Y-axis. Time is presented in X-axis: T1 = pre-training, T2 = post-training, T3 = 6-month continued practice follow-up, T4 = 1-year continued practice follow-up. Blue line = MSC; Green line = MBSR; Red line = CG. [file Image_5.JPEG]

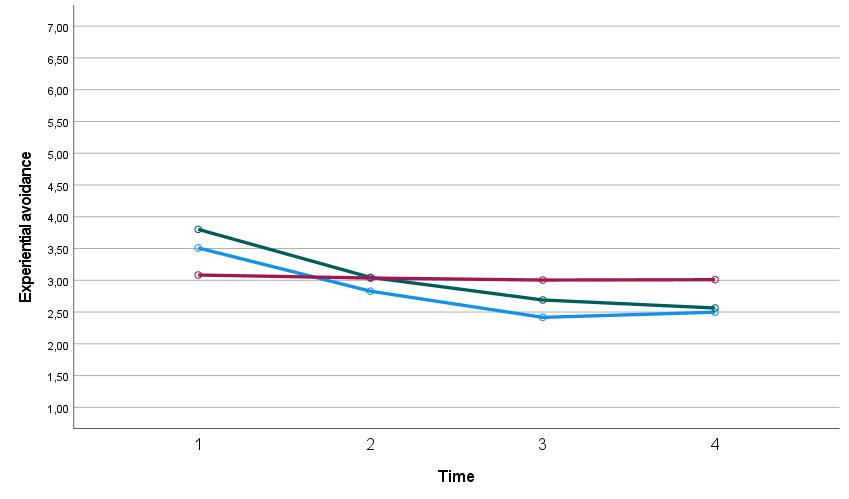

Supplement: SUPPLEMENTARY FIGURE 6 — Experiential avoidance. Estimated marginal means are presented in Y-axis. Time is presented in X-axis: T1 = pre-training, T2 = post-training, T3 = 6-month continued practice follow-up, T4 = 1-year continued practice follow-up. Blue line = MSC; Green line = MBSR; Red line = CG. [file Image_6.JPEG]

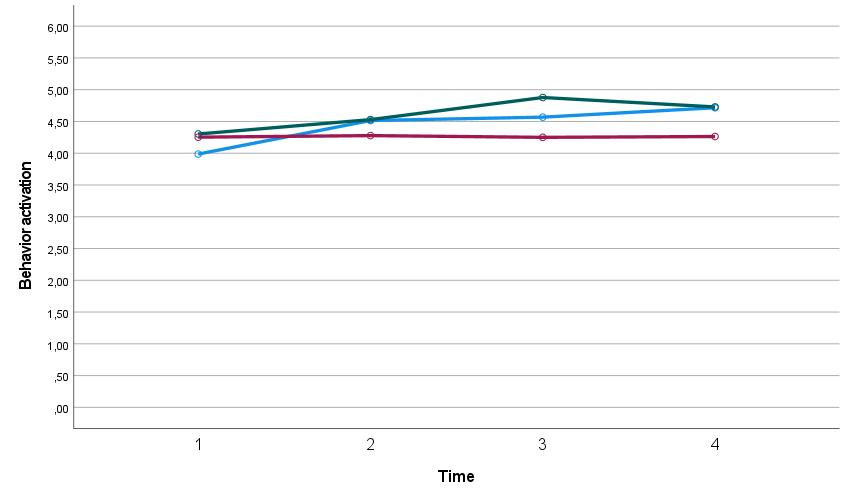

Supplement: SUPPLEMENTARY FIGURE 7 — Behaviour activation. Estimated marginal means are presented in Y-axis. Time is presented in X-axis: T1 = pre-training, T2 = post-training, T3 = 6-month continued practice follow-up, T4 = 1-year continued practice follow-up. Blue line = MSC; Green line = MBSR; Red line = CG. [file Image_7.JPEG]

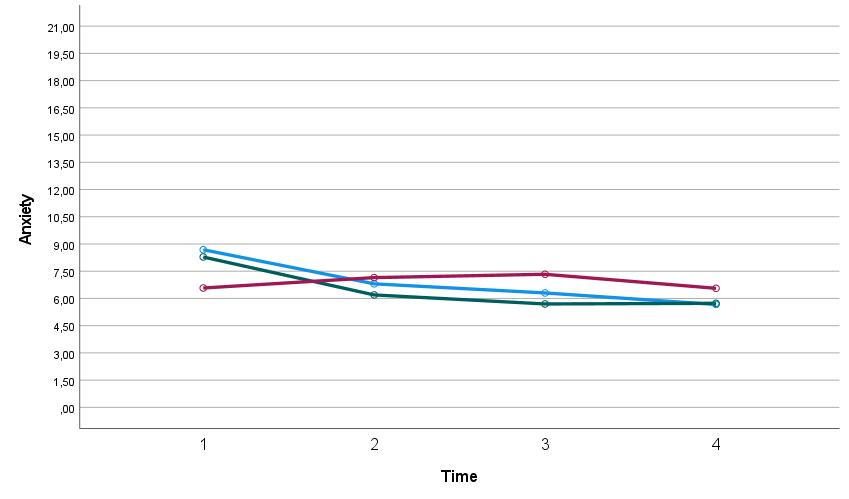

Supplement: SUPPLEMENTARY FIGURE 8 — Anxiety. Estimated marginal means are presented in Y-axis. Time is presented in X-axis: T1 = pre-training, T2 = post-training, T3 = 6-month continued practice follow-up, T4 = 1-year continued practice follow-up. Blue line = MSC; Green line = MBSR; Red line = CG. [file Image_8.JPEG]

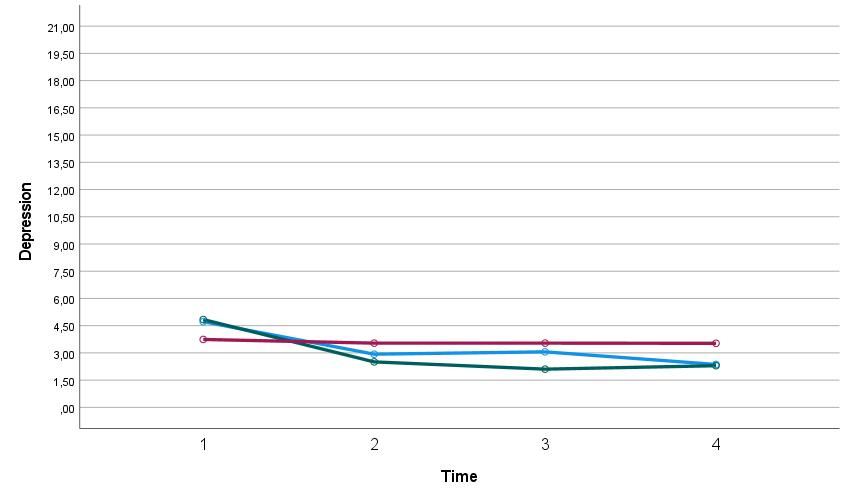

Supplement: SUPPLEMENTARY FIGURE 9 — Depression. Estimated marginal means are presented in Y-axis. Time is presented in X-axis: T1 = pre-training, T2 = post-training, T3 = 6-month continued practice follow-up, T4 = 1-year continued practice follow-up. Blue line = MSC; Green line = MBSR; Red line = CG. [file Image_9.JPEG]

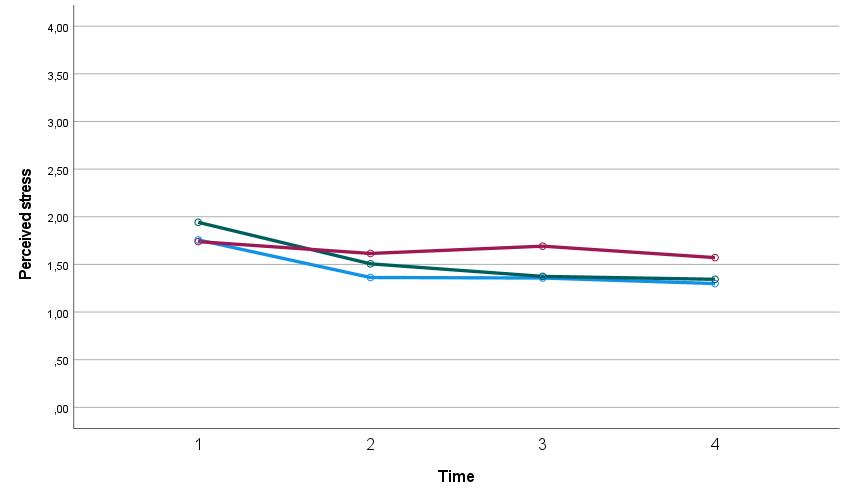

Supplement: SUPPLEMENTARY FIGURE 10 — Perceived stress. Estimated marginal means are presented in Y-axis. Time is presented in X-axis: T1 = pre-training, T2 = post-training, T3 = 6-month continued practice follow-up, T4 = 1-year continued practice follow-up. Blue line = MSC; Green line = MBSR; Red line = CG. [file Image_10.JPEG]

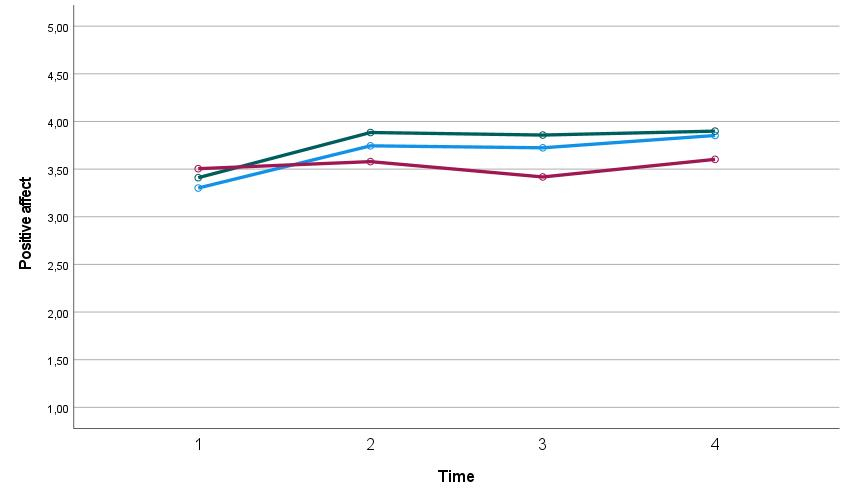

Supplement: SUPPLEMENTARY FIGURE 11 — Positive affect. Estimated marginal means are presented in Y-axis. Time is presented in X-axis: T1 = pre-training, T2 = post-training, T3 = 6-month continued practice follow-up, T4 = 1-year continued practice follow-up. Blue line = MSC; Green line = MBSR; Red line = CG. [file Image_11.JPEG]

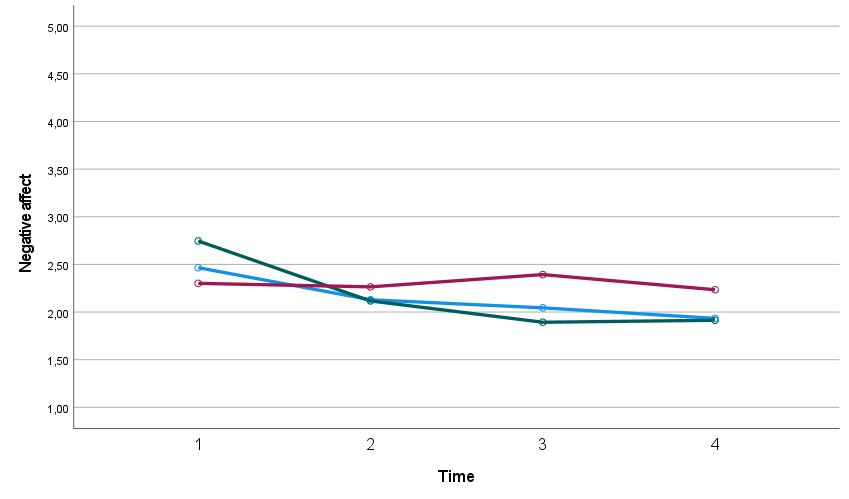

Supplement: SUPPLEMENTARY FIGURE 12 — Negative affect. Estimated marginal means are presented in Y-axis. Time is presented in X-axis: T1 = pre-training, T2 = post-training, T3 = 6-month continued practice follow-up, T4 = 1-year continued practice follow-up. Blue line = MSC; Green line = MBSR; Red line = CG. [file Image_12.JPEG]

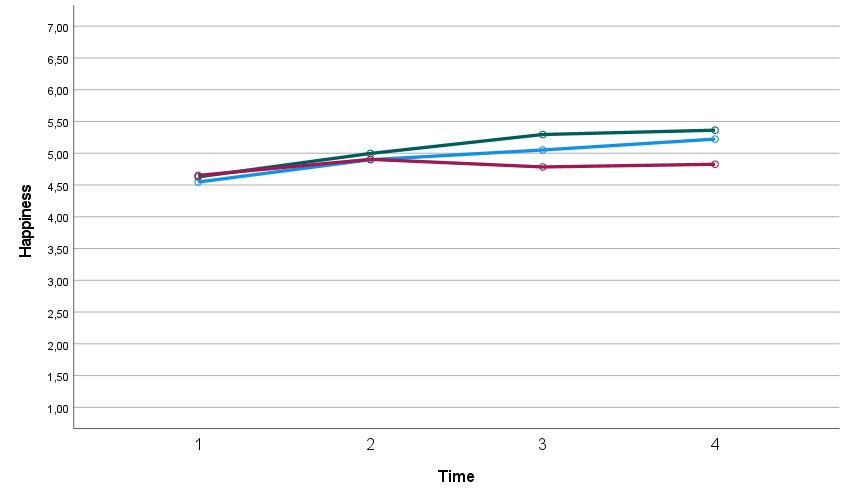

Supplement: SUPPLEMENTARY FIGURE 13 — Happiness. Estimated marginal means are presented in Y-axis. Time is presented in X-axis: T1 = pre-training, T2 = post-training, T3 = 6-month continued practice follow-up, T4 = 1-year continued practice follow-up. Blue line = MSC; Green line = MBSR; Red line = CG. [file Image_13.JPEG]

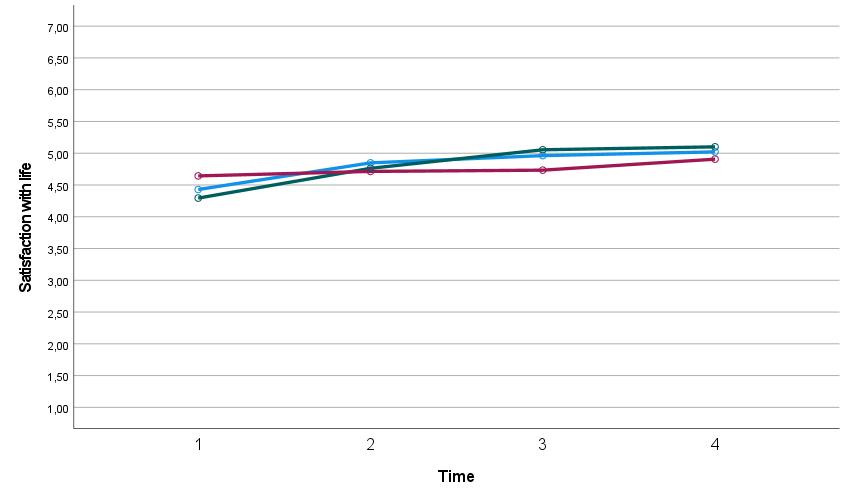

Supplement: SUPPLEMENTARY FIGURE 14 — Satisfaction with life. Estimated marginal means are presented in Y-axis. Time is presented in X-axis: T1 = pre-training, T2 = post-training, T3 = 6-month continued practice follow-up, T4 = 1-year continued practice follow-up. Blue line = MSC; Green line = MBSR; Red line = CG. [file Image_14.JPEG]
